# Solidagoic Acids L and M: Novel Antibacterial *cis*-Clerodane Diterpenoids Isolated from the Inflorescences of *Solidago gigantea* via a Bioassay-Guided Approach

**DOI:** 10.3390/antibiotics15070687

**Published:** 2026-07-14

**Authors:** Márton Baglyas, Zoltán Bozsó, Ágnes M. Móricz

**Affiliations:** 1 Plant Protection Institute, HUN-REN Centre for Agricultural Research, Fehérvári út 132–144, H-1116 Budapest, Hungary; bozso.zoltan@atk.hun-ren.hu; 2 Doctoral School, Semmelweis University, Üllői út 26, H-1085 Budapest, Hungary

**Keywords:** *Solidago gigantea* Ait., natural antibiotics, antibacterial activity, *Bacillus subtilis*, secondary metabolites, clerodane diterpenoids, phytochemicals, bioassay-guided isolation, thin-layer chromatography–direct bioautography, effect-directed analysis

## Abstract

**Background/Objectives**: Plant secondary metabolites remain an invaluable source of novel antibacterial phytochemicals in the fight against antibiotic resistance. The medicinal plant *Solidago gigantea* Ait. (giant goldenrod) is an invasive species in Europe and represents an abundant, yet largely underexplored reservoir of such bioactive compounds. The primary aim of this study was to perform a non-targeted, effect-directed screening, detection, bioassay-guided isolation, structure elucidation, and microbiological assessment of the antibacterial constituents present in the inflorescences of *S. gigantea*. **Methods**: Thin-layer chromatography coupled with direct bioautography (TLC–DB) assay using *Bacillus subtilis* was utilized for the non-targeted, effect-directed analysis of antibacterial components and the evaluation of in vitro antibacterial activity. Successive preparative flash column chromatography, semi-preparative reversed-phase high-performance liquid chromatography (RP-HPLC), and thin-layer chromatography–mass spectrometry (TLC–MS) were employed for the bioassay-guided fractionation and isolation. The structures of the isolated compounds were elucidated using one- and two-dimensional nuclear magnetic resonance (NMR) spectroscopy and high-resolution tandem mass spectrometry (HRMS/MS). The presence of known antibacterial compounds was established via reversed-phase ultra-high-performance liquid chromatography coupled with high-resolution electrospray ionization tandem mass spectrometry (RP-UHPLC–HR-ESI-MS/MS). **Results**: Two previously undescribed *cis*-clerodane diterpenoids, the isomeric solidagoic acid L (**1**) and solidagoic acid M (**2**), were isolated, identified, and characterized from the ethyl acetate extract of *S. gigantea* inflorescences. Both compounds exhibited in vitro antibacterial activity against the Gram-positive *B. subtilis*, confirmed via TLC–DB. In addition, 23 known compounds with antibacterial activity, including 17 clerodane diterpenes, four hydroxylated polyunsaturated fatty acids, and two unsaturated monoacylglycerols, were detected. All of these are reported for the first time in the inflorescences of this plant species. **Conclusions**: With further optimization, the isolated compounds may represent promising leads for antibacterial drug development. Our findings demonstrate the potential of non-targeted, bioassay-guided approaches for the discovery of novel plant-derived bioactive natural products.

## 1. Introduction

The excessive and inappropriate use of antibiotics, together with natural evolutionary processes, has contributed significantly to the global spread of antibacterial resistance [1]. The growing resistance crisis underscores the urgent need for novel antibacterial agents, particularly those with new mechanisms of action [2,3]. Plants are widely recognized as an inexhaustible source of structurally diverse secondary metabolites with a wide range of biological activities, including antibacterial effects [4]. Numerous plant-derived compounds, such as clerodane diterpenoids, have demonstrated inhibitory activity against a broad range of bacteria [5,6,7,8,9]. Consequently, the biologically active constituents of plants are promising candidates for combating multidrug-resistant microorganisms [10].

**Figure 1:**
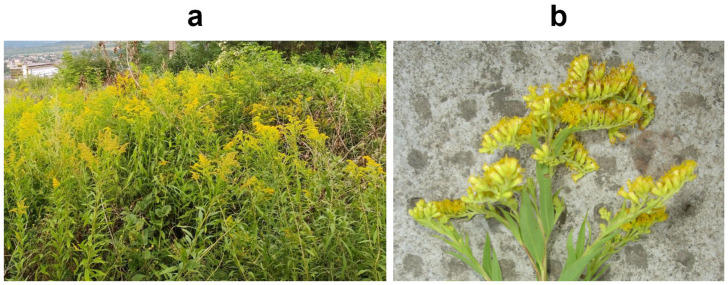
Extensive invasion of *Solidago gigantea* Ait. (giant goldenrod) in a meadow near Dorog, Hungary (**a**), and the yellow inflorescences of *S. gigantea* (**b**).

*Solidago gigantea* Ait. (giant goldenrod) (Figure 1) is a rhizomatous perennial species native to North America that has emerged as one of the most aggressive invasive weeds in Europe, owing to its rapid vegetative growth, prolific seed production, and allelopathic activity [11,12]. It has been listed as an invasive alien plant by the European and Mediterranean Plant Protection Organization (EPPO) since 2004 [13]. As invasive species continue to pose serious ecological and environmental threats, exploring their potential benefits—such as utilizing *S. gigantea* for antibiotic discovery—may offer a dual advantage: limiting their spread while identifying novel antibacterial agents. Besides its ecological significance, *S. gigantea* is also recognized as a medicinal plant. Its flowering aerial parts, together with those of *Solidago canadensis* and *Solidago virgaurea*, are officially included in the European Pharmacopoeia as *Solidaginis herba* [14]. Traditionally, these parts have been used in phytotherapy for their anti-inflammatory, diuretic, spasmolytic, and antimicrobial properties [15]. A broad range of secondary metabolites, such as flavonoids [16,17,18,19], phenolic acids [16,17,18,19], monoterpenoids [20,21], sesquiterpenoids [20,22], diterpenoids [20,23,24,25,26,27], and triterpenoids [18,28,29], were identified in *S. gigantea*. Extracts prepared from the aboveground parts of *S. gigantea* displayed significant antibacterial activity against a range of Gram-positive bacteria, including methicillin-resistant strains, and showed bacteriostatic effects, while exhibiting only low or no activity against Gram-negative bacteria [18,19,30]. Essential oil obtained from the inflorescences of *S. gigantea* demonstrated notable antibacterial activity against the opportunistic, biofilm-forming pathogens *Escherichia coli*, *Pseudomonas aeruginosa*, and methicillin-resistant *Staphylococcus aureus* (MRSA) [20]. Compounds responsible for this effect were identified as monoterpenoids, sesquiterpenoids, and diterpenoids [20,22,31]. However, several previous studies have focused on essential oils and extracts mainly obtained from the whole aerial parts of the plant rather than specifically from the inflorescences. Consequently, the antibacterial constituents of *S. gigantea* inflorescences remain insufficiently characterized, and information on their antibacterial activity remains limited.

Several bioactive clerodane diterpenoids, including antibacterial phytochemicals, have previously been isolated and characterized by our research group from the roots [32,33] and leaves [23,24] of *S. gigantea*. Building on these findings, the present study focuses on the phytochemical investigation of *S. gigantea* inflorescences as part of our ongoing search for novel plant-derived antibacterial natural products.

One of the primary objectives of this work was to screen the ethyl acetate extract of *S. gigantea* inflorescences for antibacterial compounds using a non-targeted, effect-directed thin-layer chromatography–direct bioautography (TLC–DB) assay with *Bacillus subtilis*. Additionally, the highly targeted bioassay-guided fractionation and isolation of compounds responsible for the observed antibacterial effect were achieved via successive preparative flash column chromatography and semi-preparative reversed-phase high-performance liquid chromatography (RP-HPLC). The purification process was continuously monitored by TLC–hyphenations, such as TLC with ultraviolet (TLC–UV) and fluorescence detection (TLC–FLD), TLC–*p*-anisaldehyde derivatization, TLC–mass spectrometry (TLC–MS), and TLC–DB. This was followed by the nuclear magnetic resonance (NMR) spectroscopy- and high-resolution electrospray ionization tandem mass spectrometry (HR-ESI-MS/MS)-based structure elucidation of the isolated compounds. The in vitro antibacterial activity against *B. subtilis* was assessed via TLC–DB. Furthermore, the presence of 23 selected known antibacterial compounds was also investigated in the ethyl acetate extract of *S. gigantea* inflorescences via reversed-phase ultra-high-performance liquid chromatography coupled with high-resolution electrospray ionization tandem mass spectrometry (RP-UHPLC–HR-ESI-MS/MS).

## 2. Results and Discussion

### 2.1. TLC–Direct Bioautography and Bioassay-Guided Fractionation

**Figure 2:**
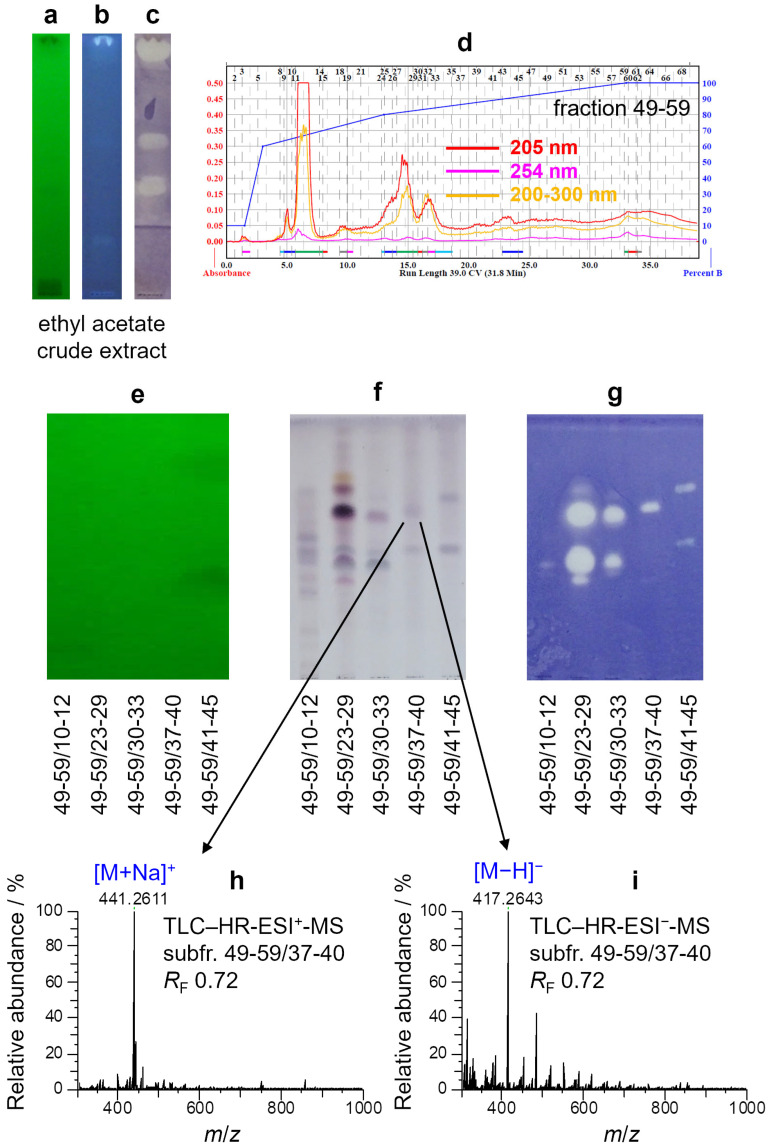
TLC chromatograms visualized at 254 nm (**a**,**e**), at 366 nm (**b**), and after derivatization with *p*-anisaldehyde reagent (**f**). TLC–*B. subtilis* bioautograms (**c**,**g**) of the ethyl acetate crude extract of *Solidago gigantea* inflorescences and pooled subfractions 49–59/10–12, 49–59/23–29, 49–59/30–33, 49–59/37–40, and 49–59/41–45, developed with the mobile phase chloroform–ethyl acetate–methanol, 15:3:2 (*V*/*V*). UV chromatograms at 205 nm (red), 254 nm (magenta), and 200–300 nm (yellow) (**d**) obtained via preparative, reversed-phase (RP) flash column chromatography of fraction 49–59 using a C_18_ column as a stationary phase with a water + 0.1% formic acid/methanol + 0.1% formic acid gradient elution (blue). TLC–HR-ESI^+^-MS (**h**) and TLC–HR-ESI^−^-MS (**i**) spectra of the antibacterial zone at *R*_F_ 0.72 of the combined subfraction 49–59/37–40.

Aiming to uncover previously undescribed antibacterial constituents in the inflorescences of *S. gigantea*, the ethyl acetate crude extract of its inflorescences was screened via TLC–UV/FLD (Figure 2a,b) and TLC–*B. subtilis* direct bioautography (DB) (Figure 2c). This non-targeted, effect-directed analysis revealed multiple inhibition zones (Figure 2c), confirming the presence of antibacterial compounds in the crude extract. For their identification, the crude extract was subjected to a bioassay-guided fractionation by preparative flash column chromatography (Figure 2d), applying normal-phase (NP) (silica gel) followed by reversed-phase (RP) (C_18_) separations to leverage the orthogonality of these stationary phases. The entire process was monitored via TLC–UV (Figure 2e), TLC–*p*-anisaldehyde derivatization (Figure 2f), TLC–DB using a *B. subtilis* antibacterial assay (Figure 2g), TLC–MS (Figure 2h,i), TLC–MS/MS, RP-HPLC–UV–ESI-MS, RP-UHPLC–HR-ESI-MS, and RP-UHPLC–HR-ESI-MS/MS. All fractions were analyzed via TLC–UV, TLC–*p*-anisaldehyde derivatization, and TLC–DB, and those with similar chromatographic fingerprints were combined. Fractions that did not produce inhibition zones in the TLC–DB assay using *B. subtilis*, indicating the absence of detectable antibacterial activity, were excluded from further fractionation.

### 2.2. Detection of Known Antibacterial Compounds via RP-UHPLC–HR-ESI-MS(/MS)

TLC–MS and TLC–MS/MS analysis of the antibacterial zones in flash chromatographic fractions suggested that the antibacterial constituents previously isolated from the roots [32,33,34] and leaves [23,24] may also be present in the inflorescences of *S. gigantea*. Therefore, a RP-UHPLC–HR-ESI-MS(/MS) method was developed for the detection and identification of these compounds using in-house reference standards, including the clerodane diterpenoids solidagoic acids A–F (**3**–**8**) and H–K (**9**–**12**), solidagodiol (**13**), solidagolactone IX (**14**), Sg1 (**15**), Sg2 (**16**), Sg3a (**17**), Sg3b (**18**), Sg4 (**19**), and the monoacylglycerols 1-linoleoyl glycerol (**20**), and 1-α-linolenoyl glycerol (**21**) (Figure S1). (Solidagoic acid G was previously isolated from the aerial parts of *Solidago virgaurea* by Starks et al. [35]; however, its occurrence was not examined in the present study due to the absence of a reference standard.) Moreover, the presence of the antibacterial oxylipins 9-hydroxy-10*E*,12*Z*-octadecadienoic acid (9-HODE, **22**, Figure S1) and 13-hydroxy-9*Z*,11*E*-octadecadienoic acid (13-HODE, **23**, Figure S1), previously isolated from the stem bark of *Ailanthus altissima* by our group [36], was also investigated. The antibacterial activity of all listed compounds was confirmed in situ using TLC–DB, with most of them validated by in vitro microplate assays [23,24,32,33,35].

Our preliminary hypothesis was confirmed, as all of the compounds listed above were detected in at least one of the flash fractions 33–35, 36–39, 40–43, 44–48, 49–59 (Table 1, Figure S2). Consequently, these compounds were not subjected to further isolation. Due to the lack of reference standards, the hydroxylated fatty acids 9-hydroxy-10*E*,12*Z*,15*Z*-octadecatrienoic acid (9-HOTrE, **24**, Figure S1) and 13-hydroxy-9*Z*,11*E*,15*Z*-octadecatrienoic acid (13-HOTrE, **25**, Figure S1) were only tentatively identified with level E confidence according to Çiçek et al. [37]. This tentative assignment was based on the precursor ion at *m*/*z* 293.2122 [M−H]^−^, the characteristic fragment ions resulting from an *α*-cleavage at *m*/*z* 171.1027 (C_9_H_15_O_3_^−^) and *m*/*z* 195.1391 (C_12_H_19_O_2_^−^) for 9-HOTrE and 13-HOTrE, respectively, and their common dehydrated fragment ion at *m*/*z* 275.2019 [M−H−H_2_O]^−^ [38] (Figure S3). Given that these compounds are naturally occurring plant metabolites, the positions and the configurations of their double bonds were presumed based on their likely biosynthetic origin as autoxidatively formed and lipoxygenase-derived products of α-linolenic acid.

In RP-UHPLC–HR-ESI-MS, the protonated molecules ([M+H]^+^) of certain clerodane diterpenes readily underwent in-source fragmentation to form dehydrated fragment ions ([M+H−H_2_O]^+^; **11**–**19**) and product ions corresponding to the loss of angelate ([M+H−C_5_H_8_O_2_]^+^; **4** and **6**) and isobutyrate moieties ([M+H−C_4_H_8_O_2_]^+^; **12**). In addition, the protonated molecules ([M+H]^+^) of certain carboxylic acids (**5**, **7**, **9**) exhibited in-source fragmentation with a loss of formic acid, yielding fragment ions [M+H−HCOOH]^+^. Beyond the commonly observed adduct ions ([M+H]^+^, [M+Na]^+^, [M+NH_4_]^+^, [M−H]^−^), these in-source fragment ions enabled more confident identification.

**Figure 3:**
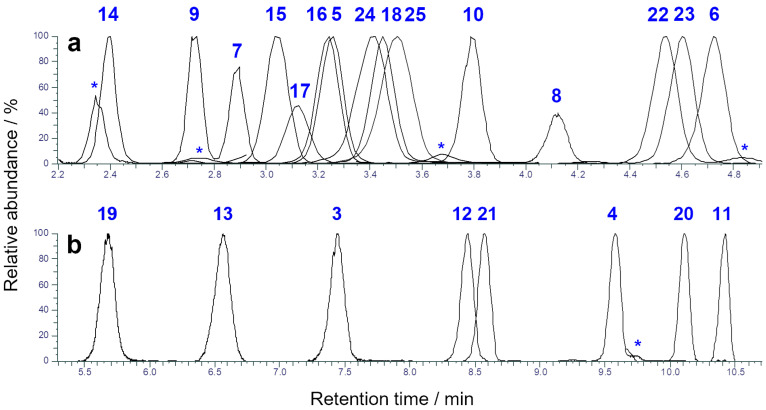
Overlaid and locally normalized extracted ion chromatograms (EIC)—first segment (**a**), second segment (**b**)—of the 23 studied, known compounds (**3**–**25**) obtained via RP-UHPLC–HR-ESI-MS/MS analysis. Retention times (*t*_R_/min): 2.40 (**14**), 2.73 (**9**), 2.90 (**7**), 3.03 (**15**), 3.13 (**17**), 3.26 (**5**), 3.30 (**16**), 3.42 (**24**), 3.45 (**18**), 3.50 (**25**), 3.79 (**10**), 4.12 (**8**), 4.54 (**22**), 4.60 (**23**), 4.72 (**6**), 5.67 (**19**), 6.57 (**13**), 7.44 (**3**), 8.44 (**12**), 8.57 (**21**), 9.58 (**4**), 10.11 (**20**), 10.42 (**11**). Precursor ions and normalized collision energies (NCE) are summarized in Table 1. Peaks denoted by an asterisk (*) indicate impurities.

In RP-UHPLC–HR-ESI-MS/MS, sodium adduct ions ([M+Na]^+^) were unsuitable as precursor ions except for **12** due to their high stability, resulting in fragment-poor MS/MS spectra. Although all solidagoic acids could be ionized in negative ionization mode, HCD fragmentation of the deprotonated molecules ([M−H]^−^) of isobutyrate (**12**) and certain angelate (**4**, **6**, **11**) esters was suboptimal. This phenomenon is attributed to the high stability of the angelate (*m*/*z* 99.0452, C_5_H_8_O_2_^−^) and isobutyrate (*m*/*z* 87.0452, C_4_H_8_O_2_^−^) ions, leading to the formation of only a single fragment ion, even at high collision energies. The RP-UHPLC–HR-ESI-MS/MS EIC chromatograms of the 23 studied, known compounds (**3**–**25**) are shown in Figure 3 in two segments for better visualization.

The detection of the 23 known compounds (**3**–**25**) in the inflorescences of *S. gigantea* demonstrates that the biosynthesis of these secondary metabolites is not restricted to vegetative organs. Their occurrence in reproductive tissues suggests that these constituents may contribute to the chemical defense of inflorescences against bacterial pathogens, thereby enhancing the protection of reproductive structures and supporting reproductive fitness.

### 2.3. Bioassay-Guided Isolation and the Antibacterial Activity of the Isolated Compounds

**Figure 4:**
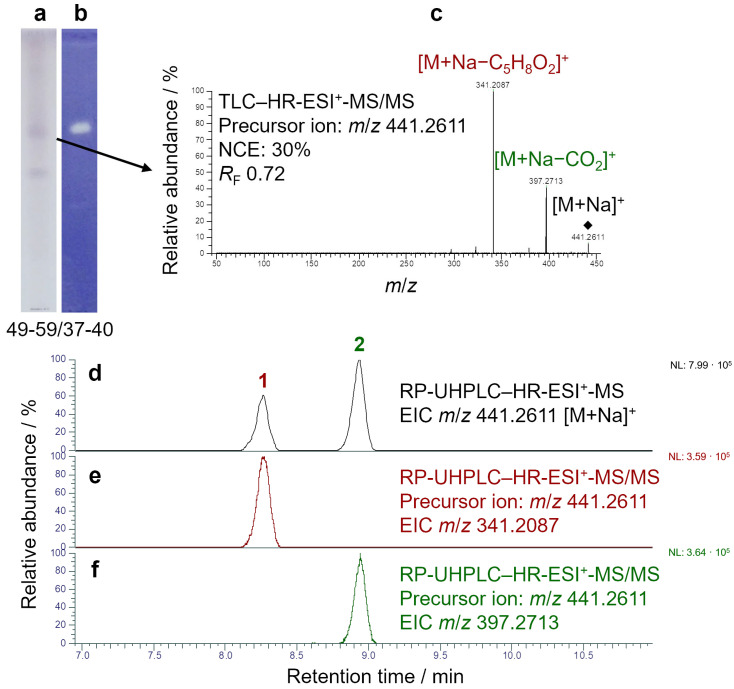
TLC chromatogram documented at white light illumination after derivatization with *p*-anisaldehyde reagent (**a**) and TLC–*B. subtilis* bioautogram (**b**) of subfraction 49–59/37–40, both developed with the mobile phase chloroform–ethyl acetate–methanol, 15:3:2 (*V*/*V*). TLC–HR-ESI^+^-MS/MS spectrum recorded at *R*_F_ 0.72 with a precursor ion at *m*/*z* 441.2611 [M+Na]^+^ and a normalized collision energy (NCE) of 30% (**c**). EIC chromatogram at *m*/*z* 441.2611 [M+Na]^+^ of the RP-UHPLC–HR-ESI^+^-MS analysis of subfraction 49–59/37–40—compound **1** (*t*_R_ = 8.26 min) and **2** (*t*_R_ = 8.94 min) ((**d**), black). EIC chromatograms at *m*/*z* 341.2087 ((**e**), dark red) and *m*/*z* 397.2713 ((**f**), green) of the RP-UHPLC–HR-ESI^+^-MS/MS analysis of subfraction 49–59/37–40 with a precursor ion at *m*/*z* 441.2611 [M+Na]^+^ and an NCE of 30%.

TLC–HR-ESI-MS analysis of the bioactive zone of subfraction 49–59/37–40 at *R*_F_ 0.72 (TLC mobile phase: chloroform–ethyl acetate–methanol 15:3:2, *V*/*V*) (Figure 4a,b) displayed ions at *m*/*z* 441.2611 [M+Na]^+^ in positive (Figure 2h) and at *m*/*z* 417.2643 [M−H]^−^ in negative ionization mode (Figure 2i), which could not be assigned to any of the compounds reported from *S. gigantea* in the literature or previously identified in our laboratory. Hence, this subfraction was selected for isolation. MS HCD fragmentation of the precursor ion at *m*/*z* 441.2611 [M+Na]^+^ with a normalized collision energy (NCE) of 30% produced two main fragment ions at *m*/*z* 341.2087 [M+Na−C_5_H_8_O_2_]^+^ and 397.2713 [M+Na−CO_2_]^+^ (Figure 4c). However, RP-UHPLC–HR-ESI-MS analysis revealed that the ion at *m*/*z* 441.2611 [M+Na]^+^ corresponds to two distinct chromatographic peaks (*t*_R_ = 8.26 and 8.94 min) (Figure 4d). Subsequent RP-UHPLC–HR-ESI-MS/MS experiment with a precursor ion at *m*/*z* 441.2611 [M+Na]^+^ and a NCE of 30% demonstrated that the fragment ion at *m*/*z* 341.2087 was yielded by compound **1**, eluting at *t*_R_ = 8.26 min (Figure 4e), and the fragment ion at *m*/*z* 397.2713 was observed for compound **2**, eluting at *t*_R_ = 8.94 min (Figure 4f). These findings indicate the presence of two isomers, compounds **1** and **2**, which co-migrated on TLC silica gel but could be resolved on a C_18_ HPLC column. This observation highlights a fundamental challenge in the phytochemical profiling of complex plant extracts. While NP TLC–DB is a convenient, cost-effective, valuable, and rapid tool for localizing antibacterial activity, TLC has limited resolving power for closely related isomers, which underscores the necessity of employing complementary, orthogonal techniques such as RP-HPLC.

**Figure 5:**
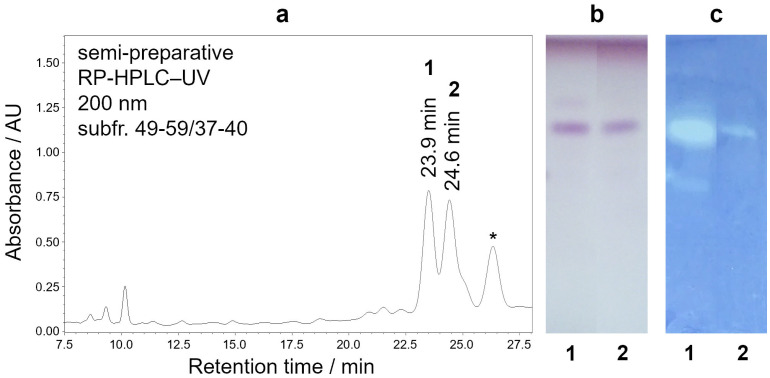
Semi-preparative RP-HPLC–UV chromatogram recorded at 200 nm during the isolation of compound **1** (1.3 mg, *t*_R_ = 23.7 min) and compound **2** (1.1 mg, *t*_R_ = 24.6 min) from subfraction 49–59/37–40 (**a**). Note that the peak denoted by an asterisk (*) at *t*_R_ = 26.4 min corresponds to chloroform (the solvent of the fraction). TLC chromatogram after derivatization with *p*-anisaldehyde reagent (**b**) and TLC–*B. subtilis* bioautogram (**c**) of the isolated compounds **1** (*R*_F_ 0.72) and **2** (*R*_F_ 0.72) with a quantity of 1 μg, both developed with the mobile phase chloroform–ethyl acetate–methanol, 15:3:2 (*V*/*V*).

To enable their unambiguous identification, the two isomers with similar retention characteristics were isolated from subfraction 49–59/37–40 by semi-preparative RP-HPLC–UV at 200 nm, affording compound **1** (1.3 mg, *t*_R_ = 23.7 min) and compound **2** (1.1 mg, *t*_R_ = 24.6 min) (Figure 5a). After derivatization with *p*-anisaldehyde, compounds **1** and **2** appeared as pink spots at *R*_F_ 0.72 on the TLC plates (Figure 5b). The in situ antibacterial activity of the isolated compounds (**1** and **2**) was confirmed against the Gram-positive *B. subtilis* bacteria with a quantity of 1 μg via TLC–DB. This was evidenced by white inhibition zones against a purplish background at *R*_F_ 0.72 for compounds **1** and **2** (TLC mobile phase: chloroform–ethyl acetate–methanol 15:3:2, *V*/*V*) (Figure 5c). Notably, the inhibition zone corresponding to compound **1** was more intense than that of compound **2** (Figure 5c), suggesting a higher antibacterial potency. Due to undesirable oxidative degradation of the samples during storage, their antibacterial activity (MIC, IC_50_) against *B. subtilis* and additional bacterial and fungal strains could not be evaluated using in vitro microplate assays.

### 2.4. Structure Elucidation

The structures of the isolated compounds (**1** and **2**) were elucidated using one- (1D) and two-dimensional (2D) NMR spectroscopy and HR-ESI-MS(/MS). The novelty of compounds **1** and **2** was verified by database searches in CAS SciFinder^®^ and Reaxys. The acquired 1D and 2D NMR (Figures S4–S10 for **1** and Figures S15–S21 for **2**) and HR-ESI-MS(/MS) spectra (Figures S11–S14 for **1** and Figures S22–S25 for **2**) are provided in the Supplementary Materials.

**Figure 6:**
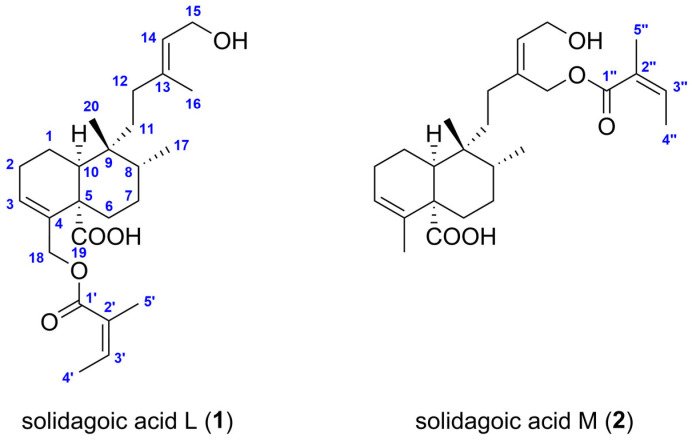
The chemical structures of the previously undescribed isolated compounds: solidagoic acid L (**1**) and solidagoic acid M (**2**) with atomic numbering (blue).

Solidagoic acid L (**1**) (Figure 6) was obtained as a white amorphous solid. Its molecular formula was established as C_25_H_38_O_5_ based on the ^13^C DEPTQ NMR spectrum and deduced from the positive (*m*/*z* 441.2611 [M+Na]^+^, calculated for C_25_H_38_O_5_Na^+^, *m*/*z* 441.2612 [M+Na]^+^, error: −0.2 ppm) and negative ion mode (*m*/*z* 417.2644 [M−H]^−^, calculated for C_25_H_37_O_5_^−^, *m*/*z* 417.2647 [M−H]^−^, error: −0.6 ppm) HR-ESI-MS spectra, demanding seven double bond equivalents (DBEs). Its ^1^H NMR spectrum (Table 2) displayed proton signals corresponding to five methyl groups at *δ*_H_ 1.98 (dq, *J* = 7.3, 1.4 Hz, 3H, H_3_-4′), *δ*_H_ 1.89 (p, *J* = 1.4 Hz, 3H, H_3_-5′), *δ*_H_ 1.66 (s, 3H, H_3_-16), *δ*_H_ 0.91 (s, 3H, H_3_-20), and *δ*_H_ 0.82 (d, *J* = 6.4 Hz, 3H, H_3_-17), three vinylic hydrogens at *δ*_H_ 6.04 (qq, *J* = 7.3, 1.4 Hz, 1H, H-3′), *δ*_H_ 5.92 (t, *J* = 4.1 Hz, 1H, H-3), and *δ*_H_ 5.40 (t, *J* = 7.1 Hz, 1H, H-14), and two oxymethylene groups at *δ*_H_ 4.50 (m, 2H, H_2_-18) and *δ*_H_ 4.16 (d, *J* = 7.1 Hz, 2H, H_2_-15). Based on ^13^C DEPTQ (Table 2), ^1^H–^13^C multiplicity-edited HSQC (edHSQC), and ^1^H–^13^C HMBC spectroscopic data, the twenty-five ^13^C resonances were assigned to five methyl groups (*δ*_C_ 26.7, 20.8, 17.0, 15.9, 15.9), six aliphatic methylene (*δ*_C_ 32.9, 30.2, 28.9, 28.0, 26.5, 19.6) and two oxygenated (*δ*_C_ 64.5, 59.4) methylene groups, two aliphatic (*δ*_C_ 42.5, 37.1) methine and three olefinic (*δ*_C_ 138.3, 128.2, 121.3) methine groups, and seven non-hydrogenated carbons, including three olefinic (*δ*_C_ 141.8, 136.0, 128.0), two aliphatic (50.1, 38.6), one ester (*δ*_C_ 167.7), one carboxylic (*δ*_C_ 178.9) carbons. Thus, the structure of compound **1** features three trisubstituted C=C double bonds, one ester, and one carboxylic moiety (altogether five DBEs and four oxygen atoms), suggesting a bicyclic molecule to account for the required seven DBEs. From five methyl, eight methylene, and five methine groups, 36 hydrogen atoms were accounted for, and the two remaining exchangeable hydrogens were attributed to one hydroxylic and one carboxylic group.

The 1D ^1^H and ^13^C NMR spectroscopic data of compound **1** closely resembled those of solidagoic acid J (**11**), a clerodane diterpenoid, previously isolated from the roots [33] and leaves [24] of *S. gigantea*. The key difference was the absence of signals (H-1″–H-5″, C-1″–C-5″) corresponding to the angeloyloxy group linked to C-16, accompanied by the upfield shifts of H-14 (*δ*_H_ 5.40 in **1** compared to *δ*_H_ 5.72 in **11**, Δ = −0.32 ppm) and H-15 (*δ*_H_ 4.16 in **1** compared to *δ*_H_ 4.26 in **11**, Δ = −0.10 ppm) and a methyl group in **1** (*δ*_H_ 1.66, *δ*_C_ 17.0) replacing the oxymethylene group (*δ*_H_ 4.80, 4.66; *δ*_C_ 61.7) in **11** connected to C-15 (Table S1). These observations suggested that compound **1** is a monoangelate derivative of the diangelate solidagoic acid J, bearing a single angeloyloxy moiety at C-18 while lacking the angeloyloxy group at C-16. This hypothesis was subsequently confirmed by detailed analysis of its 2D NMR data (see below).

**Figure 7:**
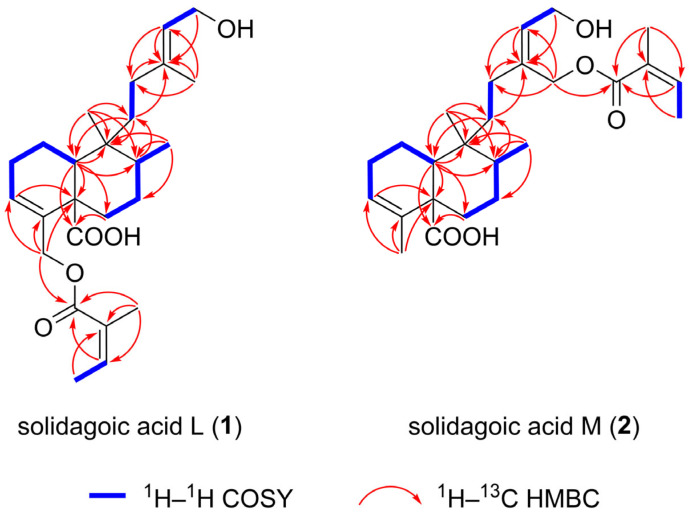
Selected ^1^H–^1^H COSY (blue) and ^1^H–^13^C HMBC (red) correlations of solidagoic acid L (**1**) and solidagoic acid M (**2**).

Five distinct spin systems (H-10/H_2_-1/H_2_-2/H-3, H_2_-6/H_2_-7/H-8/H_3_-17, H_2_-11/H_2_-12, H-14/H_2_-15, and H-3′/H_3_-4′) were evident from the ^1^H–^1^H COSY spectrum of compound **1** (Figure 7). The 6/6 fused A/B ring system (C-1–C-10) bearing two vicinal methyl groups (C-17, C-20) and one oxidized methyl group (C-18) was corroborated by the key ^1^H–^13^C HMBC correlations from H-3 to C-5, from H-10 to C-5, C-6, C-8, C-9, from H_3_-17 to C-7, C-8, C-9, from H_2_-18 to C-3, C-4, C-5, and from H_3_-20 to C-8, C-9, C-10 as well as by the spin systems H-10/H_2_-1/H_2_-2/H-3 and H_2_-6/H_2_-7/H-8/H_3_-17. The carboxylic group connected to C-5 was established by HMBC correlations from H_2_-6a, H_2_-6b, and H-10 to C-19. The structure of side chain (C-11–C-16) was supported by the HMBC correlations from H_2_-11 to C-13, from H_2_-12 to C-13, C-14, C-16, from H-14 to C-13, C-12, C-16, and from H_2_-16 to C-12, C-13, C-14 as well as by the spin systems H_2_-11/H_2_-12 and H-14/H_2_-15. The connection of the hydroxy group at C-15 was indicated by the downfield ^1^H and ^13^C NMR chemical shifts of H_2_-15 (*δ*_H_ 4.16) and C-15 (*δ*_C_ 59.4). The attachment of the side chain (C-11–C-16) to C-9 was confirmed by HMBC correlations from H_2_-11 to C-9, C-10, C-20, and from H-8, H-10, and H_3_-20 to C-11. The presence of the angeloyloxy moiety was established based on its characteristic ^1^H NMR spectroscopic data [6.04 (qq, *J* = 7.3, 1.4 Hz, 1H, H-3′), *δ*_H_ 1.98 (dq, *J* = 7.3, 1.4 Hz, 3H, H_3_-4′), *δ*_H_ 1.89 (p, *J* = 1.4 Hz, 3H, H_3_-5′)], the HMBC correlations from H-3′ to C-1′, from H_3_-4′ to C-2′, from H_3_-5′ to C-1′, C-2′, C-3′, as well as the spin system H-3′/H_3_-4′. The presence of the angeloyloxy group was further substantiated by MS/MS fragmentation analysis. On the one hand, in the positive-ion mode, the precursor ion at *m*/*z* 441.2611 [M+Na]^+^ yielded a fragment ion at *m*/*z* 341.2087 [M+Na−C_5_H_8_O_2_]^+^, corresponding to the neutral loss of an angeloyloxy moiety. On the other hand, in the negative-ion mode, the parent ion at *m*/*z* 417.2644 [M−H]^−^ resulted in the formation of a product ion at *m*/*z* 99.0452, consistent with an angelate ion (C_5_H_7_O_2_^−^). The angeloyloxy moiety is located at C-18, as evidenced by the HMBC correlation from H_2_-18 to C-1′ and by the downfield ^1^H and ^13^C NMR chemical shifts of H_2_-18 (*δ*_H_ 4.50) and C-18 (*δ*_C_ 64.5).

**Figure 8:**
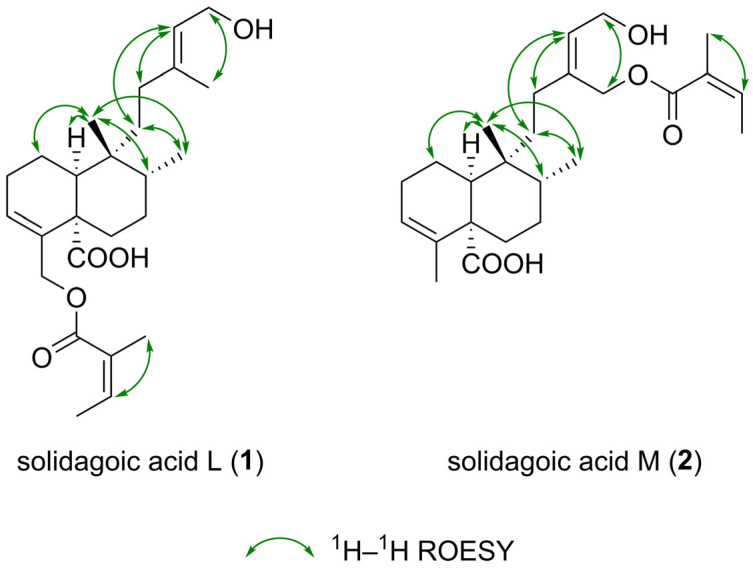
Key ^1^H–^1^H NOE correlations (green) observed in the ^1^H–^1^H ROESY spectra of solidagoic acid L (**1**) and solidagoic acid M (**2**).

The relative configuration of compound **1** was determined to be the same (*cis*–*trans* (CT)-type clerodane diterpene) as that of solidagoic acid J by comparing their ^1^H–^1^H NOE enhancements observed in ^1^H–^1^H ROESY spectra and ^1^H and ^13^C NMR chemical shifts. The *cis–trans* (CT)-type clerodane diterpene skeleton was proposed based on the ^13^C NMR chemical shift difference between C-17 and C-20 exceeding 10 ppm (∆*δ*_C-20–C-17_ = 11.2 ppm) [39,40]. The *cis*-A/B ring junction was implied by the characteristic downfield ^13^C NMR chemical shift of C-20 (*δ*_C_ 26.7) [41] and the typical downfield ^1^H NMR chemical shift of H_3_-20 (*δ*_H_ 0.91) relative to H_3_-17 (*δ*_H_ 0.82) [39]. NOE correlations between H_3_-17/H_2_-11b, H_3_-17/H_3_-20, H_3_-20/H_2_-1a, H_3_-20/H-8, and H_3_-20/H-10 established the stereochemistry and confirmed that compound **1** has a CT-type clerodane diterpene skeleton with a nonsteroidal conformation [40]. The ^1^H NMR chemical shift of H-3′ (*δ*_H_ 6.04), which closely matched the reported value for methyl angelate (*δ*_H_ 6.06) and differed significantly from that of methyl tiglate (*δ*_H_ 6.90) [42], suggested the presence of an angeloyloxy rather than a tigloyloxy group. Furthermore, the ROESY correlation between H-3′ and H_3_-5′, together with the absence of NOE interaction between H_3_-4′ and H_3_-5′, supported the *Z* configuration of the double bond (Figure 8). The ROESY spectrum exhibited cross-peaks between H_2_-11a/H-14, H_2_-11b/H-14, H_2_-12a/H-14, H_2_-12b/H-14, H_2_-15/H_2_-16a, and H_2_-15/H_2_-16b, whereas no correlations were detected between H_2_-12/H_2_-15 or H-14/H_2_-16 (Figure 8). These observations were consistent with the *E* configuration of the C-13–C-14 double bond. Thus, compound **1**, a previously undescribed *cis*-clerodane diterpenoid acid, was identified as (5S*,8R*,9R*,10S*)-18-angeloyloxy-15-hydroxycleroda-3,13*E*-dien-19-oic acid, trivially named solidagoic acid L.

Solidagoic acid M (**2**) (Figure 6) was obtained as a white amorphous solid. It gave the molecular formula C_25_H_38_O_5_, identical to that of **1**, thereby indicating that **2** is an isomer of **1**. This assignment was determined from the ^13^C DEPTQ NMR data (Table 2) and the positive-ion HR-ESI-MS peak at *m*/*z* 441.2610 [M+Na]^+^ (calculated for C_25_H_38_O_5_Na^+^, *m*/*z* 441.2612 [M+Na]^+^, error: −0.3 ppm) and the negative-ion HR-ESI-MS peak at *m*/*z* 417.2644 [M−H]^−^ (calculated for C_25_H_37_O_5_^−^, *m*/*z* 417.2647 [M−H]^−^, error: −0.6 ppm), indicating seven DBEs. The ^1^H NMR spectroscopic data (Table 2) of compound **2** closely resembled those of the isomeric compound **1**. ^1^H NMR resonances corresponding to the angeloyloxy moiety [6.10 (qq, *J* = 7.0, 1.4 Hz, 1H, H-3″), *δ*_H_ 1.98 (dq, *J* = 7.0, 1.4 Hz, 3H, H_3_-4″), *δ*_H_ 1.88 (p, *J* = 1.4 Hz, 3H, H_3_-5″)] were likewise observed. However, the resonance of H-3″ appeared slightly downfield (*δ*_H_ 6.10) compared with that in **1** (*δ*_H_ 6.04). In addition, H-3 was shifted upfield (*δ*_H_ 5.50 in **2** compared to *δ*_H_ 5.92 in **1**), whereas H-14 (*δ*_H_ 5.72 in **2** compared to *δ*_H_ 5.40 in **1**) and H-15 (*δ*_H_ 4.26 in **2** compared to *δ*_H_ 4.16 in **1**) were shifted downfield. These ^1^H NMR chemical shift differences suggested that the angeloyloxy group in compound **2** is attached at C-16 rather than at C-18 as in compound **1**. This assignment was further corroborated by the close agreement of the ^1^H NMR chemical shifts of H-14, H-15, H_2_-16a, H_2_-16b, and H-3″ with those reported for solidagoic acid J [24] (Table S1), bearing an angeloyloxy group at C-16. Moreover, HMBC correlation from H_2_-16 to C-1″ confirmed the connection of the angeloyloxy moiety at C-16, while HMBC correlations from H_3_-18 to C-3, C-4, C-5 further supported the lack of the angeloyloxy group at C-18. Further evidence for the altered position of the angeloyloxy group was obtained from the distinct MS/MS fragmentation behavior of the precursor ion at *m*/*z* 441.2611 [M+Na]^+^. In contrast to compound **1**, compound **2** did not undergo a neutral loss of an angeloyloxy group to give the fragment ion at *m*/*z* 341.2087 [M+Na−C_5_H_8_O_2_]^+^. Instead, a product ion at *m*/*z* 397.2713 [M+Na−CO_2_]^+^ was predominantly formed, consistent with the presence of a COOH group. This distinct MS fragmentation pathway further supports the different connectivity of the angeloyloxy group in compounds **1** and **2**. All the other COSY and HMBC correlations (Figure 7) for compound **2** were consistent with those observed for compound **1**, indicating that compound **2** is a monoangelate derivative of solidagoic acid J, bearing a single angeloyloxy moiety at C-16, while lacking the angeloyloxy group at C-18. Based on the NOE correlations and ^1^H and ^13^C NMR chemical shift analysis, the relative configuration of the clerodane core of compound **2** was determined to be the same (CT-type clerodane diterpene) as that of **1**. Although the same NOE enhancements were observed (Figure 8), the configuration of the C-13–C-14 double bond was determined as *Z* according to the Cahn–Ingold–Prelog (CIP) priority rules (the oxymethylene substituent has a higher priority than the adjacent methylene substituent, leading to a *Z* configuration). In contrast, for compound **1**, the methylene substituent has a higher priority than the methyl group, resulting in an *E* configuration for the C-13–C-14 double bond. Thus, compound **2**, a previously undescribed *cis*-clerodane diterpenoid acid, was identified as (5S*,8R*,9R*,10S*)-16-angeloyloxy-15-hydroxycleroda-3,13*Z*-dien-19-oic acid, trivially named solidagoic acid M. It should be noted that the specific rotation of compounds **1** and **2** could not be reliably determined due to the limited sample availability.

Both solidagoic acid L (**1**) and solidagoic acid M (**2**) are monoangelate derivatives of the diangelate solidagoic acid J (**11**) and differ solely in the position of attachment of the angeloyloxy group. This close structural relationship suggests a direct metabolic connection: compounds **1** and **2** may serve as biosynthetic precursors and intermediates undergoing sequential esterification to form compound **11**, or alternatively, they might be endogenous degradation products formed via partial hydrolysis of the diangelate parent compound. From an analytical perspective, the distinct MS fragmentation patterns and the diagnostic MS/MS fragment ions of compounds **1** and **2** enable their rapid and reliable differentiation directly at the MS level, even if co-elution occurs in complex plant extracts. Hence, these *cis*-clerodane diterpenoids can be distinguished without isolation by preparative chromatography and subsequent NMR-based structure elucidation in initial screening workflows.

Solidagoic acids A, B [26,32,34], and K [33] were reported from the roots of *S. gigantea*. Solidagoic acids C–F and H–I were isolated from the leaves of *S. gigantea* [23,24] and from the aerial parts of *Solidago virgaurea* [35]. Solidagoic acid B and C were also identified from the leaves of *Euthamia graminifolia* (formerly known as *Solidago graminifolia*) [43]. Solidagoic acid G was reported from the aerial parts of *S. virgaurea* [35] and from the leaves of *E. graminifolia* [43]. Solidagoic acid J was isolated from both the roots [33] and leaves [24] of *S. gigantea*. The present study reports the novel natural products solidagoic acid L (**1**) and solidagoic acid M (**2**) and their isolation from the inflorescences of *S. gigantea*. Additionally, in this work, the presence of solidagoic acids A–F and H–K was confirmed for the first time in the inflorescences of *S. gigantea*. The antibacterial activity of solidagoic acids A–F and H–K was confirmed against various bacteria, including *Aliivibrio fischeri*, *B. subtilis* F1276, *Bacillus spizizenii*, *Curtobacterium flaccumfaciens* pv. *flaccumfaciens*, *Clavibacter michiganensis*, *Rhodococcus fascians*, and *Staphylococcus aureus* [23,24,32,33,34,35].

In terms of structure-activity relationships (SAR), a previous study indicated that the presence of an allylic hydroxy group at C-15 is crucial for the strong antibacterial activity, and that angeloyloxy moieties might also contribute to the enhancement of this effect [33]. The present results suggest that the antibacterial activity of these *cis*-clerodane diterpenoids is influenced not only by the presence but also by the position of the angeloyloxy moieties. Since compounds **1** and **2** are monoangelate isomers with nearly identical lipophilicity, the observed difference in their antibacterial potency can be primarily attributed to the different position of the angeloyloxy group rather than altered membrane permeability. Nevertheless, this SAR analysis remains preliminary and requires further investigation to be confirmed.

## 3. Materials and Methods

### 3.1. Chemicals and Materials

Solvents and reagents were commercially available and used as received without further purification. Analytical-grade solvents (ethyl acetate, *n*-hexane, acetone, chloroform (stabilized with 5–50 ppm amylene), and methanol) and gradient-grade methanol were purchased from Molar Chemicals (Halásztelek, Hungary). HPLC-grade acetonitrile and methanol and LC-MS-grade water were supplied by Reanal (Budapest, Hungary). LC-MS-grade methanol was obtained from VWR (Radnor, PA, USA). Bidistilled water was prepared using a Vitrotech VDB-3A apparatus (Vitro-Tech-Lab Ltd., Gyál, Hungary). Ultrapure water was generated using a Millipore Direct-Q 3 UV Water Purification System (Merck, Darmstadt, Germany). Chloroform-*d* (99.80% D, water < 0.01%, stabilized with silver foil) was acquired from Eurisotop (Saint-Aubin, France). *p*-Anisaldehyde and formic acid (LC-MS grade) were obtained from Sigma-Aldrich (Burlington, MA, USA), whereas sodium chloride and tryptone (from casein, pancreatic digest) were obtained from Reanal. Acetic acid was purchased from Lach-Ner (Neratovice, Czech Republic), concentrated sulfuric acid (96%) from Carlo Erba (Milan, Italy), and 3-(4,5-dimethylthiazol-2-yl)-2,5-diphenyltetrazolium bromide (MTT) from Carl Roth (Karlsruhe, Germany). Yeast extract was acquired from Scharlab (Barcelona, Spain) and agar from Merck. *Bacillus subtilis* (F1276) was kindly provided by József Farkas (Central Food Research Institute, Budapest, Hungary).

### 3.2. Plant Material

The inflorescences of *S. gigantea* Ait. were collected near Harta, Hungary (46°41′51.5″ N 19°02′52.4″ E; altitude: 90 m a.s.l.) in August 2022. A voucher specimen (PPI-BM-SGV-2022) has been deposited at the herbarium of the Plant Protection Institute, Centre for Agricultural Research, Budapest, Hungary. The fresh plant material was air-dried at room temperature and subsequently finely powdered using a coffee grinder (Sencor SCG 2050, Sencor, Říčany, Czech Republic).

### 3.3. Extraction and Isolation

The air-dried and ground inflorescences of *S. gigantea* (150 g) were extracted via maceration three times with ethyl acetate (3 × 1000 mL, each for 72 h) at room temperature. The crude extracts were filtered (Reanal filter paper, pore size: 7–10 μm), pooled, and the solvent was evaporated in vacuo at 40 °C with a rotary evaporator (Rotavapor R-134, Büchi, Flawil, Switzerland) to afford 12.5 g of dry residue. A 10.0 g portion of this dry residue was subjected to successive preparative and semi-preparative column chromatography. The fractions from each separation step were analyzed via various TLC methods and hyphenations (UV/FLD, *p*-anisaldehyde derivatization, direct bioautography, mass spectrometry) and RP-HPLC–DAD-ESI-MS. Fractions with similar chromatographic profiles were combined.

The dry residue was fractionated via normal-phase (NP), preparative flash column chromatography (CombiFlash NextGen 300, Teledyne Isco, Lincoln, NE, USA) using a silica gel column (40 g, RediSep Rf Gold Silica, 20–40 μm, 400–632 mesh, 60 Å, Teledyne Isco) with a gradient solvent system of *n*-hexane and acetone (0.0–2.0 min, 0%; 2.0–22.0 min, 0–50%; 22.0–28.0 min, 50–100%; 28.0–35.0 min, 100% acetone; flow rate: 40 mL/min; UV detection: 200 and 210 nm) to obtain 118 fractions. Fraction 49–59 was subjected to reversed-phase (RP), preparative flash column chromatography (CombiFlash NextGen 300) on a C_18_ column (30 g, RediSep Rf Gold C18, 20–40 μm, 400–632 mesh, 100 Å, Teledyne Isco) using a gradient solvent system of water + 0.1% formic acid (A) and methanol + 0.1% formic acid (B) (0.0–1.5 min, 10% B; 1.5–3.0 min, 10–60% B; 3.0–13.0 min, 60–80% B; 13.0–33.0 min, 80–100% B; 33.0–39.0 min, 100% B; flow rate: 35 mL/min; UV detection: 205 nm) to yield 69 subfractions. Subfraction 49–59/37–40 was further purified via semi-preparative, RP high-performance liquid chromatography (RP-HPLC; LC-20 HPLC system, Shimadzu, Kyoto, Japan) at 35 °C on a Gemini C_18_ column (250 mm × 10 mm, 10 μm, 110 Å; Phenomenex, Torrance, CA, USA) using an isocratic elution with 64% B (A: 5% aqueous acetonitrile + 0.1% formic acid, B: acetonitrile + 0.1% formic acid; flow rate: 4 mL/min; UV detection: 200 nm) to provide compound **1** (1.3 mg, *t*_R_ = 23.7 min) and compound **2** (1.1 mg, *t*_R_ = 24.6 min). The HPLC system was operated and data were acquired using LabSolutions 5.72 software (Shimadzu).

### 3.4. Compound Characterization

Solidagoic acid L (**1**): White amorphous solid; ^1^H (500 MHz, CDCl_3_) and ^13^C (126 MHz, CDCl_3_) NMR spectroscopic data, see Table 2; HR-ESI-MS *m*/*z* 441.2611 [M+Na]^+^ (calculated for C_25_H_38_O_5_Na^+^, *m*/*z* 441.2612 [M+Na]^+^, error: −0.2 ppm), *m*/*z* 417.2644 [M−H]^−^ (calculated for C_25_H_37_O_5_^−^, *m*/*z* 417.2647 [M−H]^−^, error: −0.6 ppm); TLC (silica gel): *R*_F_ 0.72 (chloroform–ethyl acetate–methanol 15:3:2, *V*/*V*); color after derivatization with *p*-anisaldehyde reagent: pink.

Solidagoic acid M (**2**): White amorphous solid; ^1^H (500 MHz, CDCl_3_) and ^13^C (126 MHz, CDCl_3_) NMR spectroscopic data, see Table 2; HR-ESI-MS *m*/*z* 441.2610 [M+Na]^+^ (calculated for C_25_H_38_O_5_Na^+^, *m*/*z* 441.2612 [M+Na]^+^, error: −0.3 ppm), *m*/*z* 417.2644 [M−H]^−^ (calculated for C_25_H_37_O_5_^−^, *m*/*z* 417.2647 [M−H]^−^, error: −0.6 ppm); TLC (silica gel): *R*_F_ 0.72 (chloroform–ethyl acetate–methanol 15:3:2, *V*/*V*); color after derivatization with *p*-anisaldehyde reagent: pink.

### 3.5. TLC with UV and FLD Detection and Derivatization with p-Anisaldehyde

The crude extract, flash fractions, and isolated compounds were analyzed via normal-phase thin-layer chromatography (TLC) on aluminum- and glass-backed TLC silica gel 60 F_254_ plates (Merck). Samples were manually applied on TLC plates as 5 mm bands using a 10 µL microsyringe (Hamilton, Bonaduz, Switzerland), with track distances of 5–10 mm and an application height of 8 mm from the lower edge. The plates were developed at room temperature in a twin trough chamber (CAMAG, Muttenz, Switzerland) pre-saturated for 10 min with the mobile phase chloroform–ethyl acetate–methanol, 15:3:2 (*V*/*V*) up to a distance of 80 mm from the lower edge of the plate (corresponding to a distance of 72 mm from the applied sample to the solvent front). After development, the plates were dried using a stream of cold air from a hair dryer. Chromatograms were documented using a digital camera (Cybershot DSC-HX60, Sony, Neu-Isenberg, Germany) under a UV lamp (CAMAG) at 254 nm and 366 nm (FLD) and after *p*-anisaldehyde derivatization at visible light under white light illumination in transmittance mode (96891 Salobrena 2 LED lamp, EGLO Lux, Dunakeszi, Hungary). For *p*-anisaldehyde staining, the developed and dried TLC plates were dipped in the *p*-anisaldehyde reagent (500 μL of *p*-anisaldehyde, 10 mL of acetic acid, 100 mL of methanol, and 5 mL of concentrated sulfuric acid (96%)) and heated at 110 °C for 5 min (Advanced Hot Plate, VWR).

### 3.6. Cell Culture for TLC–Direct Bioautography

The bacterial growth during incubation was monitored by measuring the optical density at 600 nm (OD_600_) using a WPA CO 8000 Cell Density Meter (Biochrom US, Holliston, MA, USA). For preparation of the *B. subtilis* suspension used for TLC–direct bioautography (TLC–DB), 10 mL of Luria–Bertani (LB) broth (5 g/L yeast extract, 10 g/L tryptone, 10 g/L sodium chloride) was inoculated with *B. subtilis* cells from LB agar plate (5 g/L yeast extract, 10 g/L tryptone, 10 g/L sodium chloride, 15 g/L agar), and the culture was incubated in an orbital shaker (ES-20, Orbital Shaker-Incubator, SIA Biosan, Riga, Latvia) at 37 °C for 6 h. Subsequently, the bacterial suspension was diluted to OD_600_ = 0.4 with fresh LB broth and further incubated in an orbital shaker at 37 °C until an OD_600_ of 1.2 was reached. The resulting bacterial suspension was then used for immersion of the developed and dried TLC chromatoplates.

### 3.7. TLC–Direct Bioautography (TLC–Bacillus subtilis Antibacterial Assay)

The crude extract, flash fractions, and isolated compounds were studied via TLC–DB bioassay using *B. subtilis* to detect antibacterial compounds in situ, as described earlier [44,45]. Aluminum-backed TLC plates were developed and dried as outlined in Section 3.5., immersed in a *B. subtilis* cell suspension (OD_600_ = 1.2, prepared as described in Section 3.6) in LB broth to ensure uniform coverage and incubated for 2 h at 37 °C in a moistened plastic chamber (100% relative humidity) to allow bacterial growth. The plates were then stained with 3-(4,5-dimethylthiazol-2-yl)-2,5-diphenyltetrazolium bromide (MTT) by dipping into an aqueous MTT solution (1 mg/mL), followed by an additional 30 min incubation. The bioautograms were documented under visible light using a digital camera. The yellow MTT is reduced by the metabolically active bacterial cells to form purple formazan, allowing antibacterial activity to be visualized as white inhibition zones against a purple background.

### 3.8. TLC–HR-ESI-MS(/MS) and FIA–HR-ESI-MS(/MS)

TLC–HR-ESI-MS was applied to characterize the compound(s) present in the inhibition zones responsible for the observed antibacterial activity. The selected bioactive zones were eluted with methanol (MS-grade) at a flow rate of 0.2 mL/min for approximately 30 s through the oval elution head (4 mm × 2 mm) of the TLC–MS Interface 2 (CAMAG). Methanol was guided by a Vanquish Flex UHPLC system (VF-P10, Dionex Softron, Germering, Germany), and the eluate was directed into the Orbitrap Exploris 120 hybrid quadrupole-orbitrap mass spectrometer (Thermo Fisher Scientific, Bremen, Germany) equipped with a heated electrospray ionization (HESI-II) probe to acquire high-resolution mass spectra (HRMS). The spray voltage was set to +3.4 kV for positive ionization and −3.0 kV for negative ionization. The ion transfer tube and vaporizer temperatures were set to 320 °C and 250 °C, respectively. Sheath and auxiliary gases were nitrogen, with flow rates of 10 and 5 arbitrary units (au), respectively, produced by a Peak Scientific Genius XE 35 gas generator (Glasgow, UK). Full-scan mass spectra were recorded in both positive and negative ionization modes in the range of *m*/*z* 100–1000 with the EASY-IC (fluoranthene radical ions) lock mass correction in scan-to-scan mode at a resolution of 120,000 and an automatic gain control (AGC) target. A background mass spectrum obtained from the plate at the same *R*_F_ was subtracted from each spectrum recorded from the bioactive zone. TLC–HR-ESI-MS/MS measurements were carried out in higher-energy collisional dissociation (HCD) fragmentation mode with normalized collision energies (NCE) of 15–50%. A quadrupole isolation window of *m*/*z* 0.7 was applied for precursor ion selection, and tandem mass spectra were acquired at a resolution of 30,000 with the EASY-IC (fluoranthene radical ions) lock mass correction in scan-to-scan mode.

Flow injection analysis (FIA) HR-ESI-MS(/MS) spectra were recorded for the isolated compounds to determine their molecular formulae and their MS fragmentation patterns, supporting structure elucidation. Methanol was used as the carrier solvent at a flow rate of 0.2 mL/min. FIA–HR-ESI-MS and FIA–HR-ESI-MS/MS spectra were acquired with the same UHPLC–HRMS system and identical working conditions described above. Instrument operation, control, and data acquisition were carried out by Xcalibur 4.7.69 software (Thermo Fisher Scientific), and data were analyzed using FreeStyle 1.8 SP2 QF1 software (Thermo Fisher Scientific).

### 3.9. RP-UHPLC–HR-ESI-MS(/MS)

For the detection and identification of the 23 target compounds, an RP-UHPLC–HR-ESI-MS(/MS) method was developed using in-house reference standards. To mitigate matrix effects, pooled silica gel flash chromatography fractions, rather than the crude extract, were analyzed. All experiments were carried out using the UHPLC–HRMS system described in Section 3.8. Chromatographic separation was achieved on a core-shell Phenomenex Kinetex C_18_ column (100 mm × 3 mm i. d., particle size: 2.6 μm, pore size: 100 Å; Torrance, CA, USA) thermostated with still air at 35 °C. The mobile phase consisted of 0.1% (*V*/*V*) formic acid in ultrapure water (eluent A) and 0.1% (*V*/*V*) formic acid in methanol (eluent B). A flow rate of 0.5 mL/min was applied with the following gradient elution program: 0.0–5.0 min, 75% B; 5.0–15.0 min, 75–100% B; 15.0–20.0 min, 100% B, followed by a 5 min re-equilibration step (20.0–25.0 min, 75% B). The injection volume was 1 μL.

The global MS working parameters were as follows: spray voltage, +3.4 kV (positive ionization) and −3.0 kV (negative ionization); ion transfer tube temperature, 325 °C; vaporizer temperature, 350 °C; sheath gas (N_2_) flow rate, 40 au; auxiliary gas (N_2_) flow rate, 10 au; internal mass calibration with EASY-IC (fluoranthene radical ions) in scan-to-scan mode, standard AGC target. For RP-UHPLC–HR-ESI-MS analysis, full-scan mass spectra were recorded in both positive and negative ionization modes in the *m*/*z* range of 100–1000, using a resolution of 60,000 and a maximum injection time of 100 ms. For RP-UHPLC–HR-ESI-MS/MS, tandem mass spectra were acquired in HCD fragmentation mode at a resolution of 15,000 with the precursor ions (quadrupole isolation window: *m*/*z* 0.7) and normalized collision energies (NCEs) specified in Table 1, and a maximum injection time of 20 ms. Extracted ion chromatograms (EICs) were generated with mass tolerances of 2.0 ppm (RP-UHPLC–HR-ESI-MS) and 10.0 ppm (RP-UHPLC–HR-ESI-MS/MS), respectively. The target analytes were identified with level C confidence according to Çiçek et al. [37] by matching their retention times, accurate *m*/*z* values of characteristic adduct ions and in-source fragment ions in MS^1^ spectra and fragment ions in MS/MS spectra, and isotopic patterns. The identity of each compound was confirmed by comparing the relative intensity ratios of its fragment ions with those of the reference standards, providing high confidence in the assignments. However, method validation and the (semi-) quantitative determination of 23 known compounds (**3**–**25**) were beyond the scope of the present study. Instrument operation, control, and data acquisition were carried out by Xcalibur 4.7.69 software (Thermo Fisher Scientific), and data were analyzed using FreeStyle 1.8 SP2 QF1 software (Thermo Fisher Scientific).

### 3.10. NMR Spectroscopy

The isolated compounds (**1** and **2**) were dissolved in chloroform-*d* (CDCl_3_) and transferred to standard 5 mm NMR tubes. All NMR spectra were acquired on a Bruker AVANCE III 500 (^1^H: 500.1 MHz, ^13^C: 125.8 MHz; 11.7 T) spectrometer equipped with a 5 mm triple-resonance, *z*-gradient cryoprobe (CP TCI 500S2 H-C/N-D-05 Z) (Bruker Corporation, Billerica, MA, USA) at 296 K. Instrument control and data acquisition were performed using Bruker TopSpin software (version 3.5). All spectra were acquired using the standard pulse sequences available in the software library. ^1^H and ^13^C NMR chemical shifts are reported on the delta (*δ*) scale as parts per million (ppm), referenced to the NMR solvent signals (CHCl_3_ residual peak at *δ*_H_ = 7.26 ppm and CDCl_3_ at *δ*_C_ = 77.16 ppm). Signal multiplicities are designated as follows: s—singlet; br s—broad singlet; d—doublet; br d—broad doublet; t—triplet; p—pentet; m—multiplet; dt—doublet of triplets; td—triplet of doublets; dq—doublet of quartets; qq—quartet of quartets. ^1^H–^1^H spin–spin coupling constants (*J*) are given in hertz (Hz). Structure elucidation and complete ^1^H and ^13^C resonance assignments were established from ^1^H–^1^H scalar spin–spin connectivities, direct ^1^H–^13^C, long-range ^1^H–^13^C, and ^1^H–^1^H dipolar couplings using conventional one-dimensional (1D) ^1^H (*zg*) and ^13^C DEPTQ (*deptqsp*) as well as two-dimensional (2D) homonuclear ^1^H–^1^H COSY (*cosygpqf*), ^1^H–^1^H ROESY (*roesyph.2*, mixing time: 300 ms), and heteronuclear ^1^H–^13^C multiplicity-edited HSQC (edHSQC, *hsqcedetgpsisp2.3*, optimized for ^1^*J*_C–H_ = 145 Hz) and ^1^H–^13^C HMBC (*hmbcetgpl3nd*, optimized for *^n^J*_C–H_ = 8 Hz) experiments.

## 4. Conclusions

A non-targeted, effect-directed phytochemical study of the ethyl acetate extract of *Solidago gigantea* Ait. (giant goldenrod) inflorescences led to the detection, bioassay-guided fractionation, isolation, structure elucidation, and characterization of two previously undescribed *cis*-clerodane diterpenes, solidagoic acid L (**1**) and solidagoic acid M (**2**). Their in vitro antibacterial activity against the Gram-positive *Bacillus subtilis* bacteria was confirmed via thin-layer chromatography combined with direct bioautography (TLC–DB), highlighting their potential as plant-derived antibiotics. The present study also expands the current phytochemical and biological understanding of *S. gigantea* by reporting for the first time the presence of 23 known antibacterial compounds, including clerodane diterpenes, monoacylglycerols, and hydroxylated polyunsaturated fatty acids, in its inflorescences. Our results not only enrich the structural diversity of plant-derived clerodane diterpenes but also underscore the potential of *S. gigantea* as a source of antibacterial compounds. Furthermore, they emphasize the value of non-targeted, effect-directed, bioassay-guided approaches involving TLC–DB, mass spectrometry, and column chromatography for the detection, isolation, and identification of previously undescribed bioactive natural products from plants. Compared with conventional trial-and-error methodologies, the proposed workflow offers a more cost-effective, time-efficient, and reliable strategy for discovering novel antibiotics.

With proper structural optimization, these secondary metabolites could serve as valuable leads for the development of novel antibacterial agents. Future studies can focus on the comprehensive biological evaluation of compounds **1** and **2**, including the determination of their in vitro antibacterial and antifungal activities against a broader spectrum of phytopathogenic microorganisms, the assessment of their efficacy under in planta conditions, and the evaluation of their cytotoxicity and safety profile. Moreover, future research can explore the potential utilization of the invasive plant *S. gigantea* as a sustainable source of bioactive compounds by evaluating both crude extracts and the two isolated secondary metabolites for potential applications in agriculture, including formulation development and feasibility assessment as plant protection products.

## Figures and Tables

**Table 1:** Retention times (*t*_R_), characteristic ions along with their assignment in RP-UHPLC–HR-ESI-MS, the selected precursor ions for RP-UHPLC–HR-ESI-MS/MS analysis and the corresponding optimized normalized collision energies (NCE) and product ions of the 23 studied compounds (**3**–**25**).

Compound	*t*_R_/min	Characteristic Ions in UHPLC–MS (*m*/*z*)	Precursor Ion for UHPLC–MS/MS (*m*/*z*)	Product Ions (*m*/*z*)	Normalized Collision Energy/%
solidagoic acid A (**3**)	7.44	317.2111 [M+H]^+^, 339.1931 [M+Na]^+^, 315.1966 [M−H]^−^	317.2111 [M+H]^+^	299.2006	15
				271.2056	20
				253.1951	25
solidagoic acid B (**4**)	9.58	315.1955 [M+H−C_5_H_8_O_2_]^+^, 437.2298 [M+Na]^+^, 413.2333 [M−H]^−^	315.1955 [M+H−C_5_H_8_O_2_]^+^	269.1900	25
				259.1329	25
				175.1117	35
solidagoic acid C (**5**)	3.26	287.2006 [M+H−HCOOH]^+^, 333.2060 [M+H]^+^, 355.1880 [M+Na]^+^, 331.1915 [M−H]^−^	331.1915 [M−H]^−^	287.2017	25
				259.2067	40
solidagoic acid D (**6**)	4.72	331.1904 [M+H−C_5_H_8_O_2_]^+^, 431.2428 [M+H]^+^, 448.2694 [M+NH_4_]^+^, 453.2248 [M+Na]^+^, 429.2283 [M−H]^−^	448.2694 [M+NH_4_]^+^	331.1904	10
				285.1849	20
solidagoic acid E (**7**)	2.90	303.1955 [M+H−HCOOH]^+^, 371.1829 [M+Na]^+^, 347.1864 [M−H]^−^	347.1864 [M−H]^−^	259.2067	30
				257.1911	35
solidagoic acid F (**8**)	4.12	464.2643 [M+NH_4_]^+^, 469.2197 [M+Na]^+^, 445.2232 [M−H]^−^	445.2232 [M−H]^−^	345.1707	15
				301.1809	25
				99.0452	30
solidagoic acid H (**9**)	2.73	303.1955 [M+H−HCOOH]^+^, 371.1829 [M+Na]^+^, 347.1864 [M−H]^−^	347.1864 [M−H]^−^	267.1754	30
				259.2067	30
				257.1911	35
solidagoic acid I (**10**)	3.79	464.2643 [M+NH_4_]^+^, 469.2197 [M+Na]^+^, 445.2232 [M−H]^−^	445.2232 [M−H]^−^	345.1707	15
				301.1809	25
				99.0452	30
solidagoic acid J (**11**)	10.42	499.3054 [M+H−H_2_O]^+^ 534.3425 [M+NH_4_]^+^, 539.2979 [M+Na]^+^, 515.3014 [M−H]^−^	534.3425 [M+NH_4_]^+^	399.2530	10
				299.2006	15
solidagoic acid K (**12**)	8.44	315.1955 [M+H−C_4_H_8_O_2_]^+^, 425.2298 [M+Na]^+^, 401.2333 [M−H]^−^	425.2298 [M+Na]^+^	337.1774	25
			401.2333 [M−H]^−^	87.0452	20
solidagodiol (**13**)	6.57	387.2893 [M+H−H_2_O]^+^, 405.2999 [M+H]^+^, 427.2819 [M+Na]^+^	427.2819 [M+Na]^+^	327.2295	25
solidagolactone IX (**14**)	2.40	315.1955 [M+H−H_2_O]^+^, 355.1880 [M+Na]^+^	315.1955 [M+H−H_2_O]^+^	297.1849	15
				255.1380	25
				241.1223	25
Sg1 (**15**)	3.03	301.2162 [M+H−H_2_O]^+^, 341.2087 [M+Na]^+^	301.2162 [M+H−H_2_O]^+^	283.2056	20
				255.2107	15
				207.1743	30
Sg2 (**16**)	3.24	287.2006 [M+H−CO_2_]^+^, 333.2060 [M+H−H_2_O]^+^, 355.1880 [M+Na]^+^	315.1955 [M+H−H_2_O]^+^	297.1847	20
				269.1900	25
Sg3a (**17**)	3.13	297.1849 [M+H−H_2_O]^+^, 315.1955 [M+H]^+^, 337.1774 [M+Na]^+^	315.1955 [M+H]^+^	279.1743	20
				269.1900	20
				251.1794	25
Sg3b (**18**)	3.45	297.1849 [M+H−H_2_O]^+^, 315.1955 [M+H]^+^, 337.1774 [M+Na]^+^	315.1955 [M+H]^+^	269.1900	20
				251.1794	25
				233.1536	20
				215.1430	30
Sg4 (**19**)	5.67	299.2006 [M+H−H_2_O]^+^, 339.1931 [M+Na]^+^	299.2006 [M+H−H_2_O]^+^	281.1900	20
				263.1794	20
				253.1951	30
				239.1430	30
1-linoleoyl glycerol (**20**)	10.11	355.2843 [M+H]^+^, 377.2662 [M+Na]^+^	355.2843 [M+H]^+^	263.2369	15
				245.2264	15
1-α-linolenoyl glycerol (**21**)	8.57	353.2686 [M+H]^+^, 375.2506 [M+Na]^+^,	353.2686 [M+H]^+^	261.2213	20
				243.2107	20
9-HODE (**22**)	4.54	319.2244 [M+Na]^+^, 295.2279 [M−H]^−^	295.2279 [M−H]^−^	277.2173	30
				171.1027	30
13-HODE (**23**)	4.60	319.2244 [M+Na]^+^, 295.2279 [M−H]^−^	295.2279 [M−H]^−^	277.2173	30
				195.1391	35
9-HOTrE (**24**) ^a^	3.42	317.2087 [M+Na]^+^, 293.2122 [M−H]^−^	293.2122 [M−H]^−^	275.2017	20
				171.1027	25
13-HOTrE (**25**) ^a^	3.50	317.2087 [M+Na]^+^, 293.2122 [M−H]^−^	293.2122 [M−H]^−^	275.2017	20
				195.1391	30

^a^ tentative identification.

**Table 2:** ^1^H NMR (500 MHz, CDCl_3_) and ^13^C NMR (126 MHz, CDCl_3_) spectroscopic data for the isolated compounds (**1** and **2**) in chloroform-*d* (*δ* in ppm, *J* in Hz).

Position	Solidagoic Acid L (1)		Solidagoic Acid M (2)	
	*δ*_H_ (ppm), Multiplicity, *J* (Hz)	*δ*_C_ (ppm), Type	*δ*_H_ (ppm), Multiplicity, *J* (Hz)	*δ*_C_ (ppm), Type
1a	1.76, m	19.6, CH_2_	1.73, m	19.7, CH_2_
1b	1.54 ^a^		1.52, m	
2	2.18, m	26.5, CH_2_	2.08, m	26.5, CH_2_
3	5.92, t (4.1)	128.2, CH	5.50, br s	123.3, CH
4	–	136.0, C	–	136.5, C
5	–	50.1, C	–	50.9, C
6a	2.40, dt (13.8, 3.1)	30.2, CH_2_	2.32 ^a^	29.4, CH_2_
6b	1.53 ^a^		1.42, td (13.6, 4.9)	
7a	1.68, td (12.9, 3.9)	28.0, CH_2_	1.66 ^a^	28.0, CH_2_
7b	1.35 ^a^		1.31, m	
8	1.65, m	37.1, CH	1.65 ^a^	37.1, CH
9	–	38.6, C	–	38.7, C
10	2.35 ^a^	42.5, CH	2.27 ^a^	42.6, CH
11a	1.68, m	28.9, CH_2_	1.61 ^a^	30.3, CH_2_
11b	1.33 ^a^		1.19, td (13.5, 4.8)	
12a	2.14, m	32.9, CH_2_	2.27 ^a^	29.8, CH_2_
12b	1.82, m		1.95, td (13.5, 4.8)	
13	–	141.8, C	–	139.2, C
14	5.40, t (7.1)	121.3, CH	5.72, t (7.0)	129.4, CH
15	4.16, d (7.1)	59.4, CH_2_	4.26, br d (7.0)	58.9, CH_2_
16a	1.66, s	17.0, CH_3_	4.80, d (12.2)	61.4, CH_2_
16b			4.67, d (12.2)	
17	0.82, d (6.4)	15.9, CH_3_	0.79, d (6.3)	15.9, CH_3_
18	4.50, m	64.5, CH_2_	1.58, m	19.1, CH_3_
19	–	178.9, C	–	179.1, C
20	0.91, s	26.7, CH_3_	0.92, s	27.0, CH_3_
1′	–	167.7, C		
2′	–	128.0, C		
3′	6.04, qq (7.3, 1.4)	138.3, CH		
4′	1.98, dq (7.3, 1.4)	15.9, CH_3_		
5′	1.89, p (1.4)	20.8, CH_3_		
1″			–	168.6 ^b^, C
2″			–	127.7 ^b^, C
3″			6.10, qq (7.0, 1.4)	139.4, CH
4″			1.98, dq (7.0, 1.4)	16.1, CH_3_
5″			1.88, p (1.4)	20.8, CH_3_

^a^ Multiplicity and coupling constant(s) not reported due to overlapping signals. ^b^ Chemical shift determined from the HMBC spectrum.

## Data Availability

The original contributions presented in this study are included in the article and Supplementary Material. Further inquiries can be directed to the corresponding authors.

## Supplementary Materials

The following supporting information can be downloaded at: https://www.mdpi.com/article/10.3390/antibiotics15070687/s1. Reference [24] was cited in the Supplementary Materials.
