# Supplementary material for: Solidagoic Acids L and M: Novel Antibacterial cis-Clerodane Diterpenoids Isolated from the Inflorescences of Solidago gigantea via a Bioassay-Guided Approach
Source: Antibiotics (Basel). 2026 Jul 14;15(7):687. doi: 10.3390/antibiotics15070687 (PMC13405152; doi:10.3390/antibiotics15070687)
Supplement: Supplementary file 1 [file antibiotics-15-00687-s001.zip › antibiotics-4405298-supplementary.pdf]

Supporting Information

Solidagoic Acids L and M: Novel Antibacterial *cis*-Clerodane Diterpenoids Isolated from the Inflorescences of *Solidago gigantea* via a Bioassay-Guided Approach

Márton Baglyas[1,2,*], Zoltán Bozsó[1], Ágnes M. Móricz[1,2,*]

[1]Plant Protection Institute, HUN-REN Centre for Agricultural Research, Fehérvári út 132–144, H-1116 Budapest, Hungary

[2]Doctoral School, Semmelweis University, Üllői út 26, H-1085 Budapest, Hungary

*Correspondence: baglyas.marton@phd.semmelweis.hu (M.B.); moricz.agnes@atk.hun-ren.hu (Á.M.M.)

Table of contents

No.	Legend	Page
**Table S1.**	Comparison of the ^{1}H (500 MHz) and ^{13}C NMR (126 MHz) spectroscopic data in chloroform-*d* ($CDCl_3$) of the previously undescribed solidagoic acid L (**1**) and solidagoic acid M (**2**), and the known solidagoic acid J (**11**)	S-4
**Figure S1.**	The chemical structures of the 23 known, studied compounds (**3**–**25**).	S-5
**Figure S2.**	An example for the comparison of the EIC chromatograms at *m*/*z* 299.2006 (dark red, NCE: 15%) and at *m*/*z* 399.2530 (black, NCE: 10%) (mass tolerance: 10.0 ppm) of the RP-UHPLC–HR-ESI^{+}-MS/MS analysis of the sample (fraction 49–59) (**a**) and the reference standard (compound **11**) (**b**) with a precursor ion at *m*/*z* 534.3425 $[M+NH_4]^+$.	S-6
**Figure S3.**	The normalized EIC chromatograms at *m*/*z* 171.1027 ($C_9H_{15}O_3^-$; **a**, black; NCE: 25%), at *m*/*z* 195.1391 ($C_{12}H_{19}O_2^-$; **b**, dark red; NCE: 30%), and at *m*/*z* 275.2017 $[M-H-H_2O]^-$ (**c**, green; NCE: 20%) (mass tolerance: 10.0 ppm) of the RP-UHPLC–HR-ESI^{-}-MS/MS analysis of flash fraction 44–48 with a precursor ion at *m*/*z* 293.2122 $[M-H]^-$ and a NCE of 20%. The fragment ions at *m*/*z* 171.1027 and 195.1391 are characteristic for compound **24** (t_R = 3.32 min) and **25** (t_R = 3.41 min), respectively, while the product ion at *m*/*z* 275.2017 is a common dehydrated fragment ion shared by both compounds.	S-7
**Figure S4.**	^{1}H NMR spectrum of solidagoic acid L (**1**) (500 MHz, $CDCl_3$).	S-7
**Figure S5.**	^{13}C DEPTQ NMR spectrum of solidagoic acid L (**1**) (126 MHz, $CDCl_3$).	S-8
**Figure S6.**	^{1}H–^{1}H COSY NMR spectrum of solidagoic acid L (**1**) (500 MHz, $CDCl_3$).	S-8
**Figure S7.**	^{1}H–^{13}C edHSQC NMR spectrum of solidagoic acid L (**1**) (500/126 MHz, $CDCl_3$).	S-9
**Figure S8.**	^{1}H–^{13}C edHSQC NMR spectrum of solidagoic acid L (**1**) (500/126 MHz, $CDCl_3$) – aliphatic region (δ_C 44.0–14.5).	S-9
**Figure S9.**	^{1}H–^{13}C HMBC NMR spectrum of solidagoic acid L (**1**) (500/126 MHz, $CDCl_3$).	S-10
**Figure S10.**	^{1}H–^{1}H ROESY NMR spectrum of solidagoic acid L (**1**) (500 MHz, $CDCl_3$).	S-10
**Figure S11.**	HR-ESI^{+}-MS spectrum of solidagoic acid L (**1**).	S-11
**Figure S12.**	HR-ESI^{+}-MS/MS of solidagoic acid L (**1**) with a normalized HCD collision energy of 25%.	S-11
**Figure S13.**	HR-ESI^{-}-MS spectrum of solidagoic acid L (**1**).	S-11
**Figure S14.**	HR-ESI^{-}-MS/MS of solidagoic acid L (**1**) with a normalized HCD collision energy of 20%.	S-12
**Figure S15.**	^{1}H NMR spectrum of solidagoic acid M (**2**) (500 MHz, $CDCl_3$).	S-12
**Figure S16.**	^{13}C DEPTQ NMR spectrum of solidagoic acid M (**2**) (126 MHz, $CDCl_3$).	S-13
**Figure S17.**	^{1}H–^{1}H COSY NMR spectrum of solidagoic acid M (**2**) (500 MHz, $CDCl_3$).	S-13
**Figure S18.**	^{1}H–^{13}C edHSQC NMR spectrum of solidagoic acid M (**2**) (500/126 MHz, $CDCl_3$).	S-14

**Figure S19.**	^{1}H–^{13}C edHSQC NMR spectrum of solidagoic acid M (**2**) (500/126 MHz, $CDCl_3$) – aliphatic region (δ_C 44.0–14.5).	S-14
**Figure S20.**	^{1}H–^{13}C HMBC NMR spectrum of solidagoic acid M (**2**) (500/126 MHz, $CDCl_3$).	S-15
**Figure S21.**	^{1}H–^{1}H ROESY NMR spectrum of solidagoic acid M (**2**) (500 MHz, $CDCl_3$).	S-15
**Figure S22.**	HR-ESI^{+}-MS spectrum of solidagoic acid M (**2**).	S-16
**Figure S23.**	HR-ESI^{+}-MS/MS spectrum of solidagoic acid M (**2**) with a normalized HCD collision energy of 25%.	S-16
**Figure S24.**	HR-ESI^{-}-MS spectrum of solidagoic acid M (**2**).	S-16
**Figure S25.**	HR-ESI^{-}-MS/MS spectrum of solidagoic acid M (**2**) with a normalized HCD collision energy of 20%.	S-17

Table S1. Comparison of the ^{1}H (500 MHz) and ^{13}C NMR (126 MHz) spectroscopic data in chloroform-*d* ($CDCl_3$) of the previously undescribed solidagoic acid L (**1**) and solidagoic acid M (**2**), and the known solidagoic acid J (**11**).

Position	Solidagoic acid L (1) (in this publication)		Solidagoic acid J (11)*		Solidagoic acid M (2) (in this publication)	
	δ_H (ppm), Multiplicity, *J* (Hz)	δ_C (ppm), Type	δ_H (ppm), Multiplicity, *J* (Hz)	δ_C (ppm), Type	δ_H (ppm), Multiplicity, *J* (Hz)	δ_C (ppm), Type
1a	1.76, m	19.6, CH_2	1.75, m	19.6, CH_2	1.73, m	19.7, CH_2
1b	1.54[a]		1.54[a]		1.52, m	
2	2.18, m	26.5, CH_2	2.17, m	26.5, CH_2	2.08, m	26.5, CH_2
3	5.92, t (4.1)	128.2, CH	5.91, t (4.0)	128.1, CH	5.50, br s	123.3, CH
4	–	136.0, C	–	136.2, C	–	136.5, C
5	–	50.1, C	–	50.0, C	–	50.9, C
6a	2.40, dt (13.8, 3.1)	30.2, CH_2	2.41, dt (13.9, 2.8)	30.1, CH_2	2.32[a]	29.4, CH_2
6b	1.53[a]		1.51[a]		1.42, td (13.6, 4.9)	
7a	1.68, td (12.9, 3.9)	28.0, CH_2	1.68[a]	28.0, CH_2	1.66[a]	28.0, CH_2
7b	1.35[a]		1.33, m		1.31, m	
8	1.65, m	37.1, CH	1.65[a]	37.0, CH	1.65[a]	37.1, CH
9	–	38.6, C	–	38.7, C	–	38.7, C
10	2.35[a]	42.5, CH	2.32, dd (12.9, 1.5)	42.6, CH	2.27[a]	42.6, CH
11a	1.45, m	28.9, CH_2	1.59[a]	30.0, CH_2	1.61[a]	30.3, CH_2
11b	1.33[a]		1.22, td (13.7, 4.8)		1.19, td (13.5, 4.8)	
12a	2.14, m	32.9, CH_2	2.26, td (14.3, 2.4)	29.4, CH_2	2.27[a]	29.8, CH_2
12b	1.82, m		1.94, td (13.6, 4.7)		1.95, td (13.5, 4.8)	
13	–	141.8, C	–	139.1, C	–	139.2, C
14	5.40, t (7.1)	121.3, CH	5.72, t (7.1)	128.7, CH	5.72, t (7.0)	129.4, CH
15	4.16, d (7.1)	59.4, CH_2	4.26, d (7.1)	58.7, CH_2	4.26, br d (7.0)	58.9, CH_2
16a	1.66, s	17.0, CH_3	4.80, d (12.3)	61.7, CH_2	4.80, d (12.2)	61.4, CH_2
16b			4.66, d (12.3)		4.67, d (12.2)	
17	0.82, d (6.4)	15.9, CH_3	0.79, d (6.4)	15.8, CH_3	0.79, d (6.3)	15.9, CH_3
18	4.50, m	64.5, CH_2	4.51, m	64.5, CH_2	1.58, m	19.1, CH_3
19	–	178.9, C	–	178.8, C	–	179.1, C
20	0.91, s	26.7, CH_3	0.92, s	27.0, CH_3	0.92, s	27.0, CH_3
1′	–	167.7, C	–	167.7, C		
2′	–	128.0, C	–	128.0, C		
3′	6.04, qq (7.3, 1.4)	138.3, CH	6.04, qq (7.2, 1.3)	138.3, CH		
4′	1.98, dq (7.3, 1.4)	15.9, CH_3	1.97[a]	15.9, CH_3		
5′	1.89, p (1.4)	20.8, CH_3	1.88[a]	20.8, CH_3		
1″			–	168.7, C	–	168.6, C
2″			–	127.7, C	–	127.7, C
3″			6.10, qq (7.2, 1.3)	139.3, CH	6.10, qq (7.0, 1.4)	139.4, CH
4″			1.97[a]	16.0, CH_3	1.98, dq (7.0, 1.4)	16.1, CH_3
5″			1.86[a]	20.7, CH_3	1.88, p (1.4)	20.8, CH_3

[a] Multiplicity and coupling constant(s) not reported due to overlapping signals.

*Reference: Baglyas, M.; Ott, P.G.; Bozsó, Z.; Schwarczinger, I.; Bakonyi, J.; Dlauchy, D.; Darcsi, A.; Varga, S.; Móricz, Á.M. Bioassay-Guided Isolation of *cis*-Clerodane Diterpenoids and Monoglycerides from the Leaves of *Solidago gigantea* and Their Antimicrobial Activities. *Plants* **2025**, *14*, 2152, doi:10.3390/plants14142152.

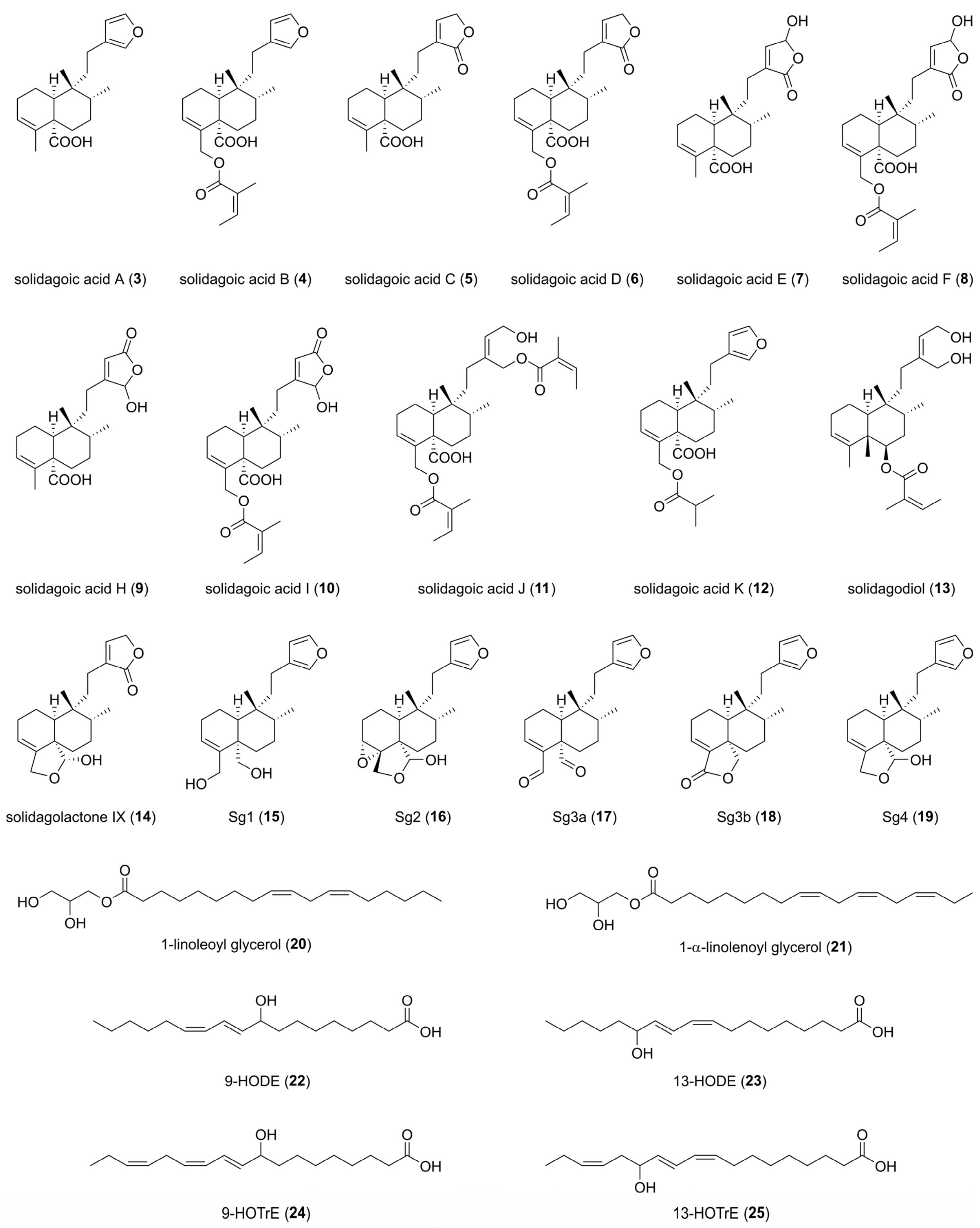

Figure S1. The chemical structures of the 23 known, studied compounds (**3**–**25**).

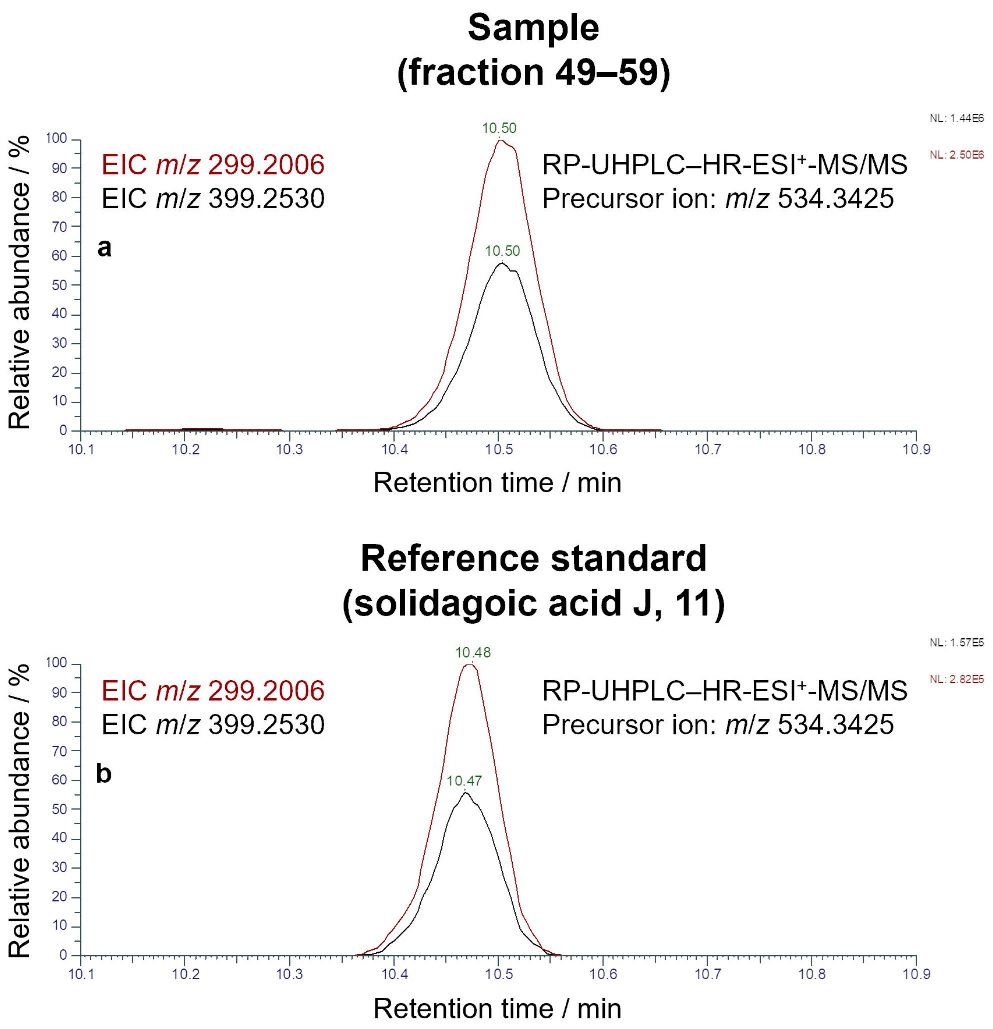

Figure S2. An example for the comparison of the EIC chromatograms at *m*/*z* 299.2006 (dark red, NCE: 15%) and at *m*/*z* 399.2530 (black, NCE: 10%) (mass tolerance: 10.0 ppm) of the RP-UHPLC–HR-ESI$^+$-MS/MS analysis of the sample (fraction 49–59) (**a**) and the reference standard (compound **11**) (**b**) with a precursor ion at *m*/*z* 534.3425 $[M+NH_4]^+$.

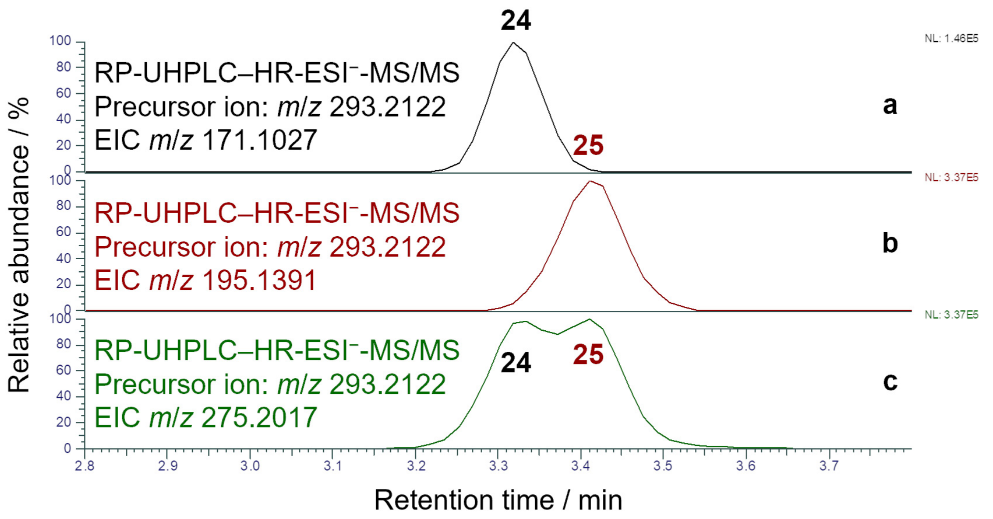

Figure S3. The normalized EIC chromatograms at *m/z* 171.1027 ($C_9H_{15}O_3^-$; **a**, black; NCE: 25%), at *m/z* 195.1391 ($C_{12}H_{19}O_2^-$; **b**, dark red; NCE: 30%), and at *m/z* 275.2017 $[M-H-H_2O]^-$ (**c**, green; NCE: 20%) (mass tolerance: 10.0 ppm) of the RP-UHPLC–HR-ESI$^-$-MS/MS analysis of flash fraction 44–48 with a precursor ion at *m/z* 293.2122 $[M-H]^-$ and a NCE of 20%. The fragment ions at *m/z* 171.1027 and 195.1391 are characteristic for compound **24** (t_R = 3.32 min) and **25** (t_R = 3.41 min), respectively, while the product ion at *m/z* 275.2017 is a common dehydrated fragment ion shared by both compounds.

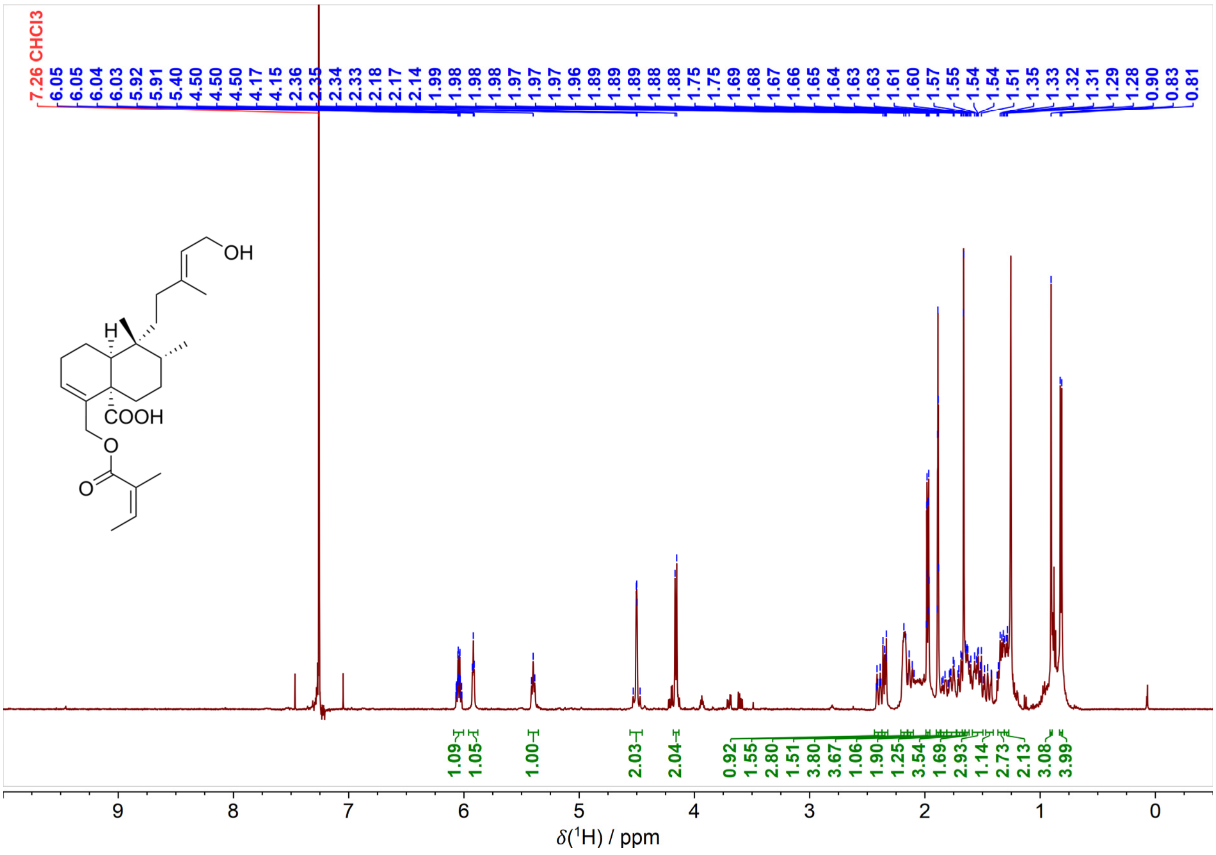

Figure S4. ¹H NMR spectrum of solidagoic acid L (**1**) (500 MHz, $CDCl_3$).

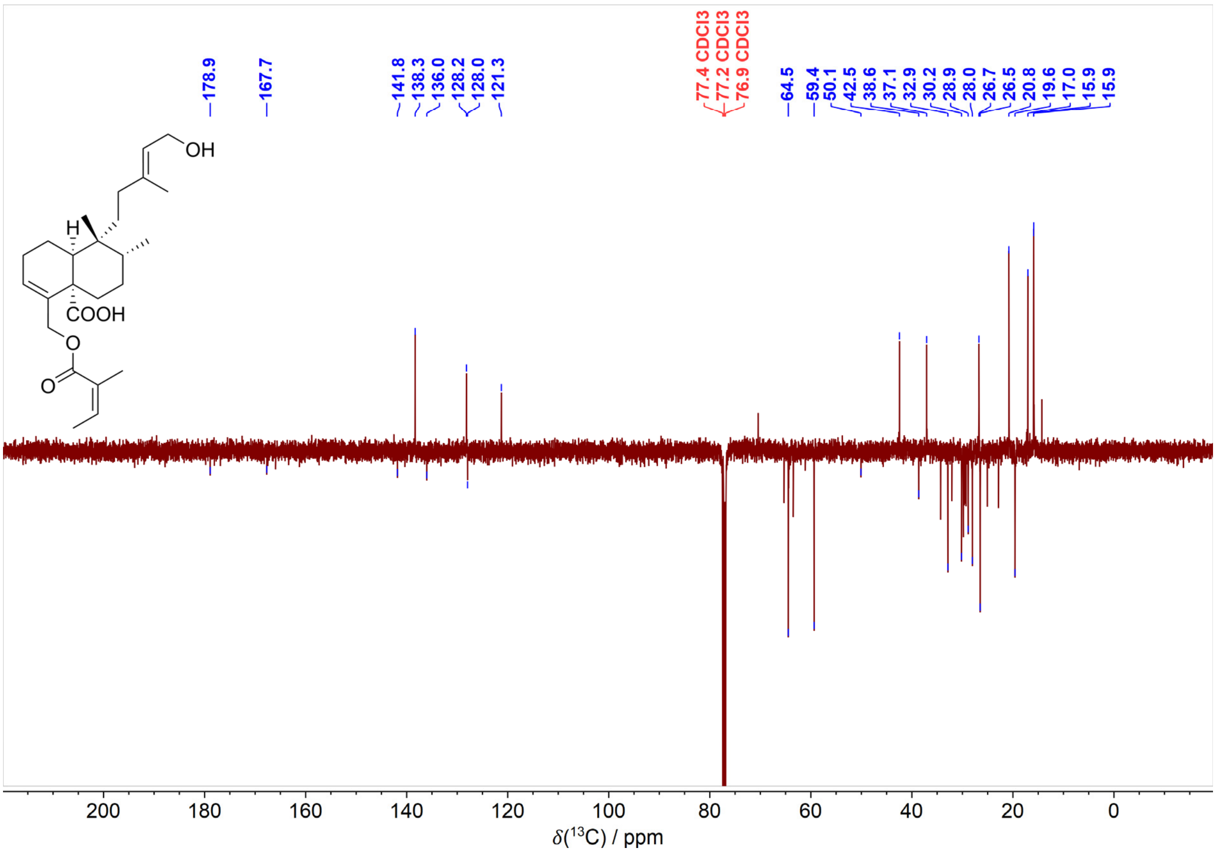

Figure S5. ^{13}C DEPTQ NMR spectrum of solidagoic acid L (**1**) (126 MHz, $CDCl_3$).

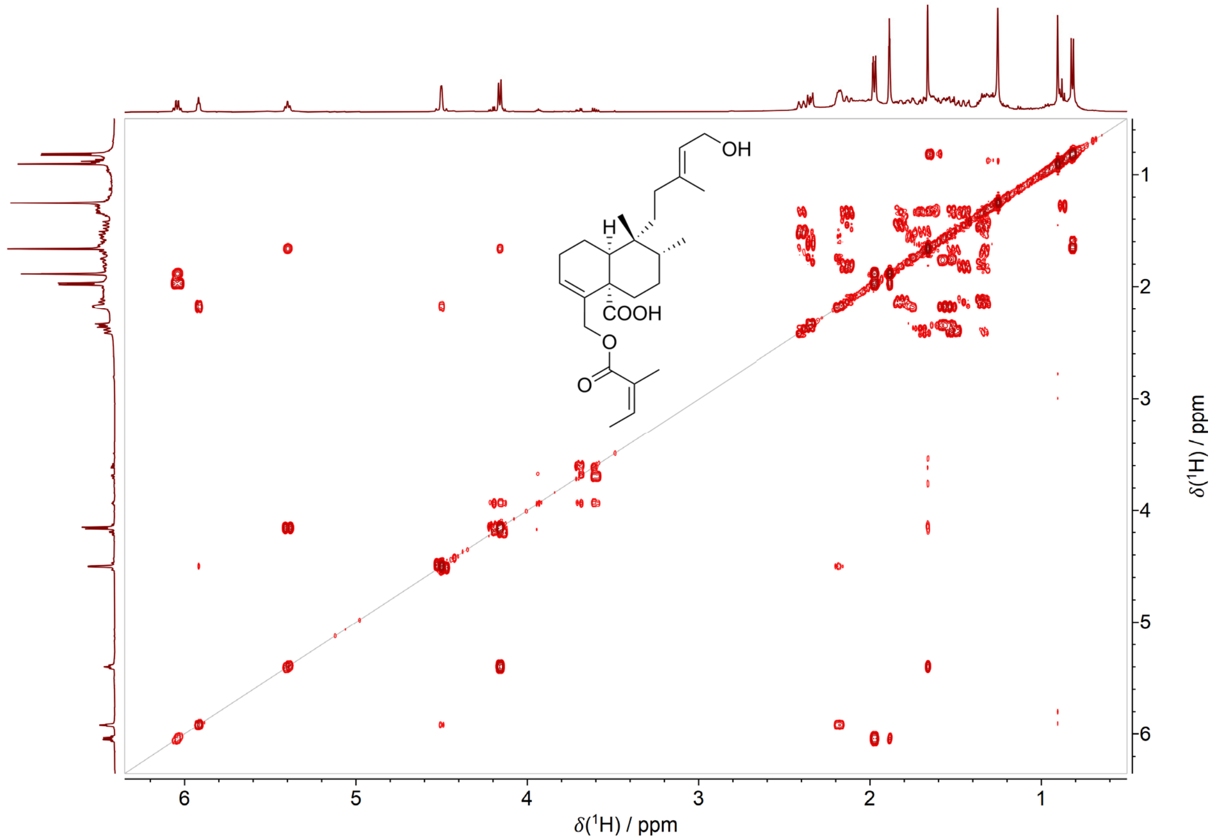

Figure S6. ^{1}H–^{1}H COSY NMR spectrum of solidagoic acid L (**1**) (500 MHz, $CDCl_3$).

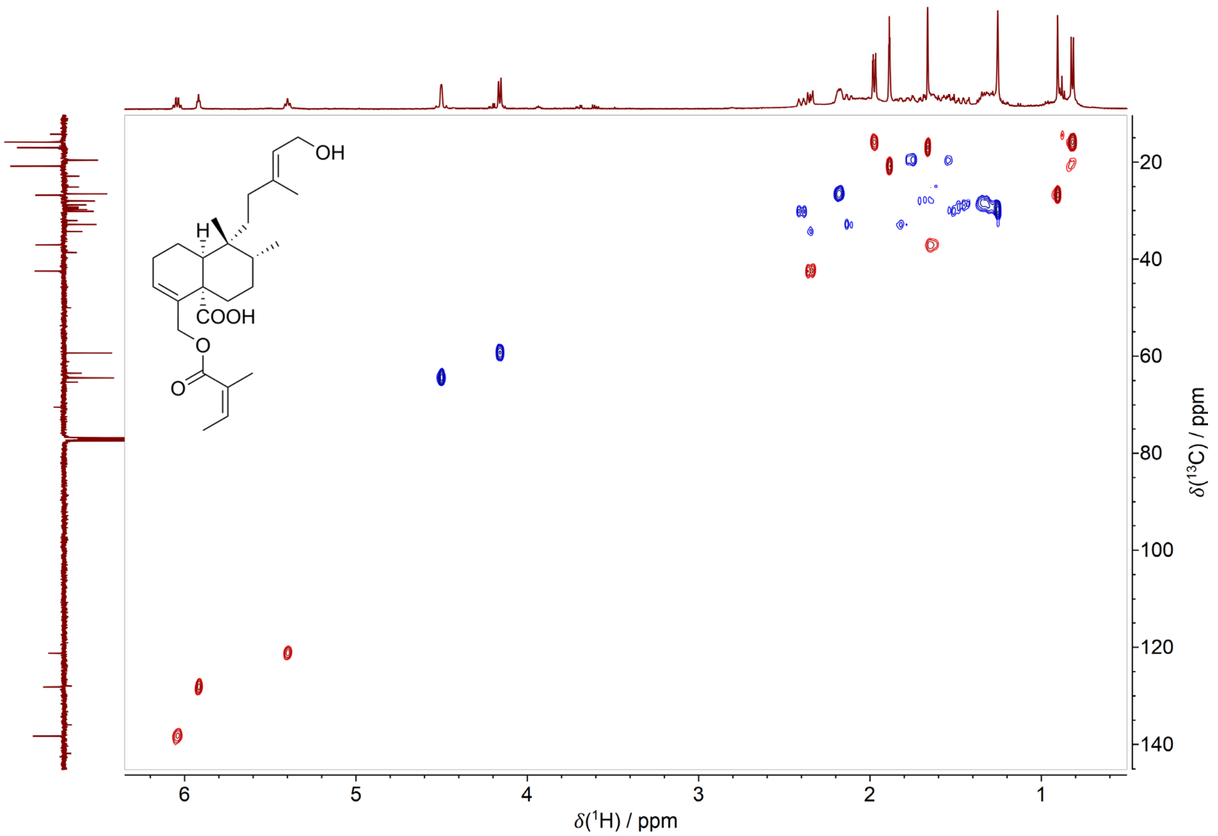

Figure S7. ^{1}H–^{13}C edHSQC NMR spectrum of solidagoic acid L (**1**) (500/126 MHz, $CDCl_3$).

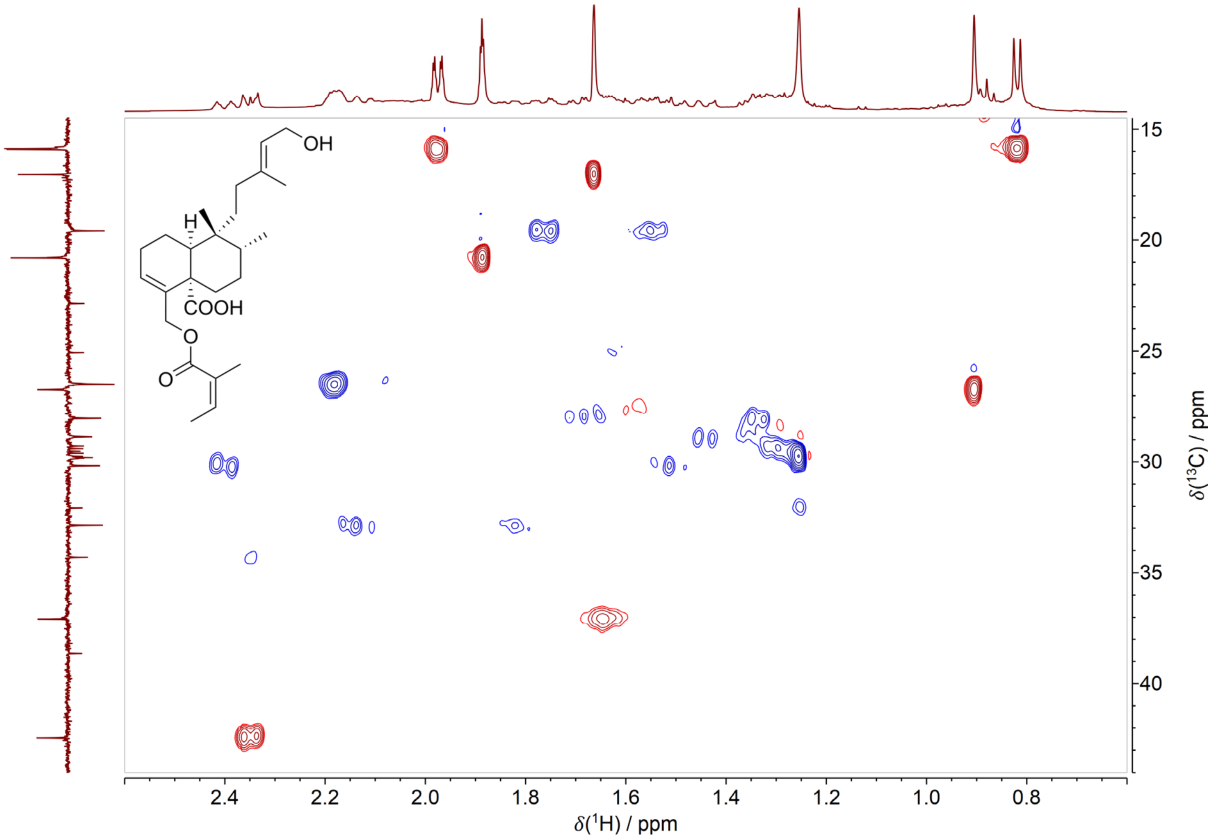

Figure S8. ^{1}H–^{13}C edHSQC NMR spectrum of solidagoic acid L (**1**) (500/126 MHz, $CDCl_3$) – aliphatic region (δ_C 44.0–14.5).

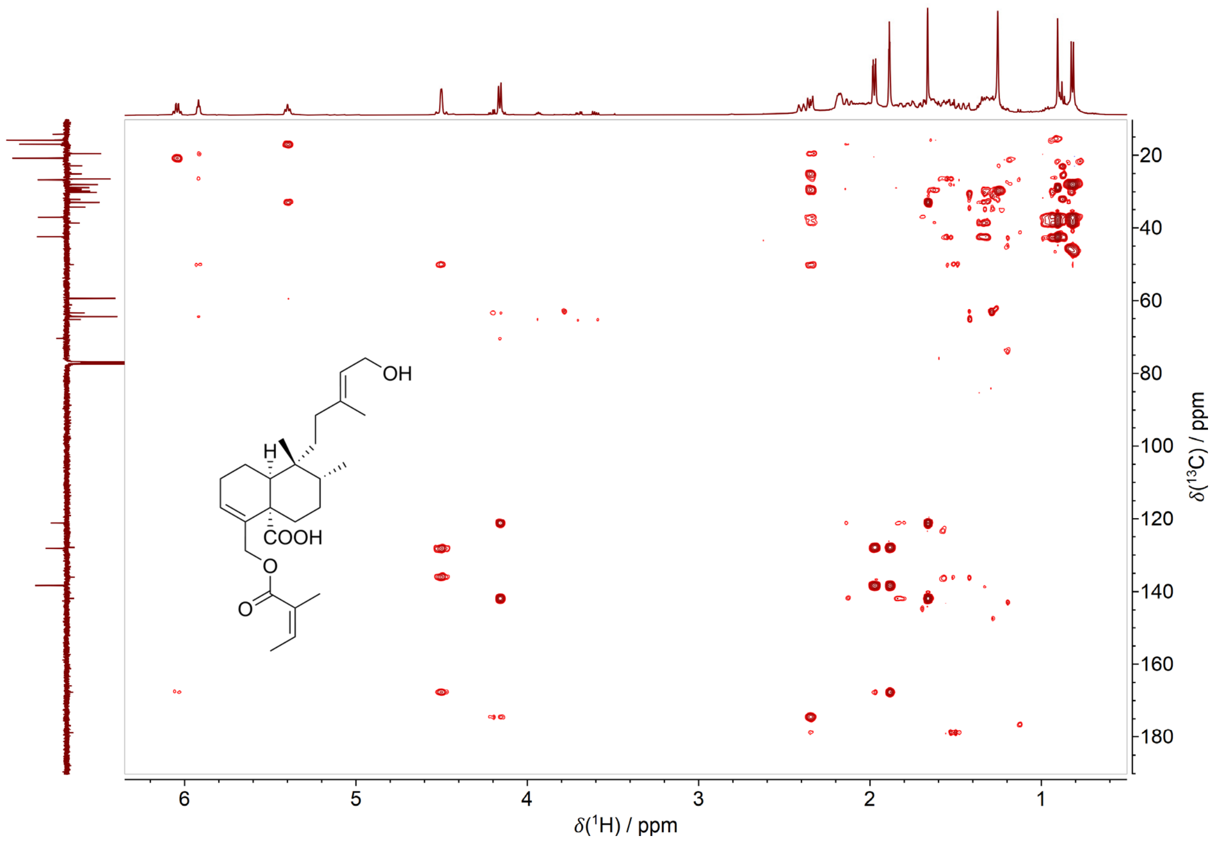

Figure S9. ¹H–¹³C HMBC NMR spectrum of solidagoic acid L (**1**) (500/126 MHz, CDCl₃).

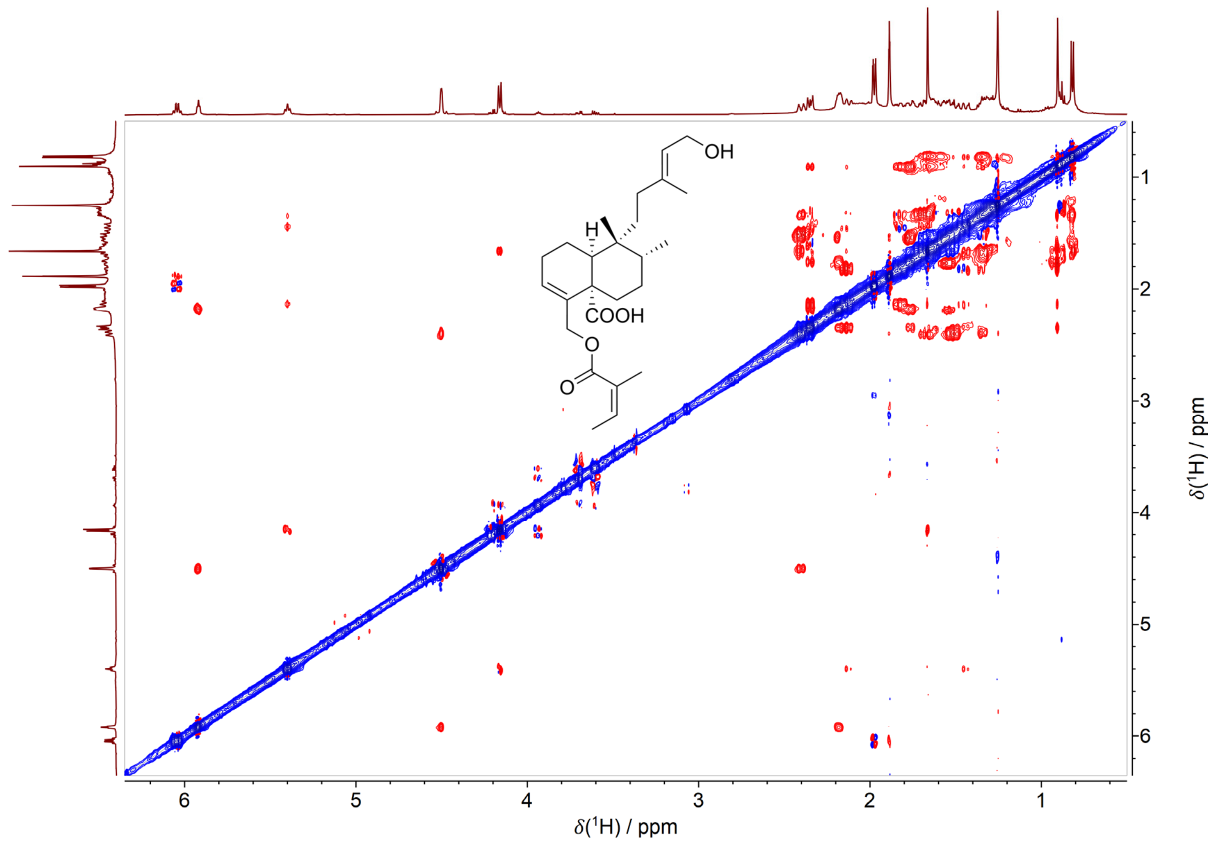

Figure S10. ¹H–¹H ROESY NMR spectrum of solidagoic acid L (**1**) (500 MHz, CDCl₃).

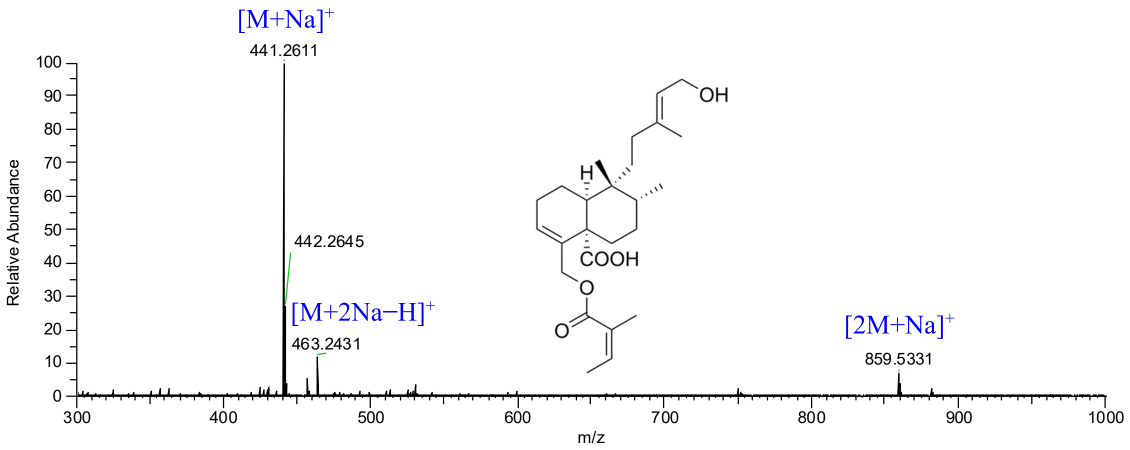

Figure S11. HR-ESI+-MS spectrum of solidagoic acid L (**1**), *m/z* 441.2611 $[M+Na]^+$ (calculated for $C_{25}H_{38}O_5Na^+$, *m/z* 441.2612 $[M+Na]^+$, error: −0.2 ppm).

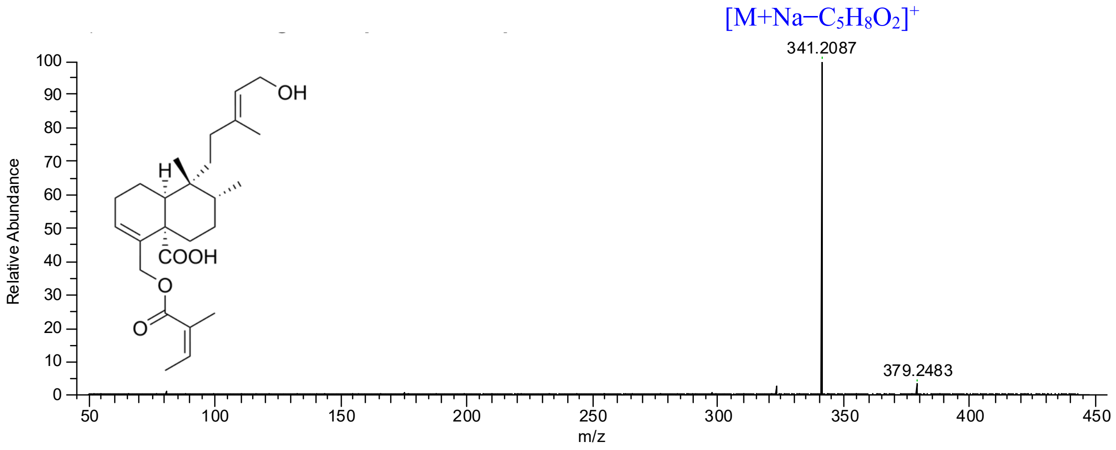

Figure S12. HR-ESI+-MS/MS spectrum of solidagoic acid L (**1**) with a normalized HCD collision energy of 25%. Precursor ion: *m/z* 441.2611 $[M+Na]^+$, $C_{25}H_{38}O_5Na^+$.

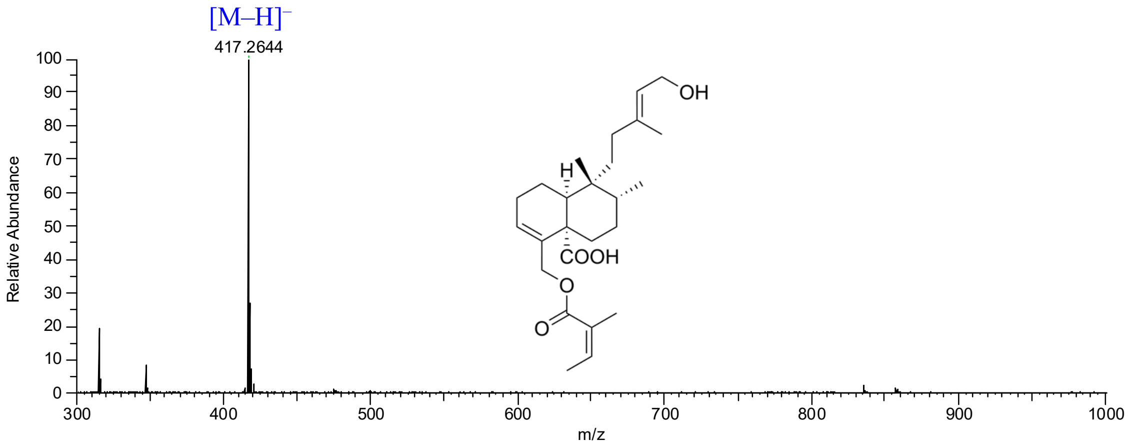

Figure S13. HR-ESI−-MS spectrum of solidagoic acid L (**1**), *m/z* 417.2644 $[M-H]^-$ (calculated for $C_{25}H_{37}O_5^-$, *m/z* 417.2647 $[M-H]^-$, error: −0.6 ppm).

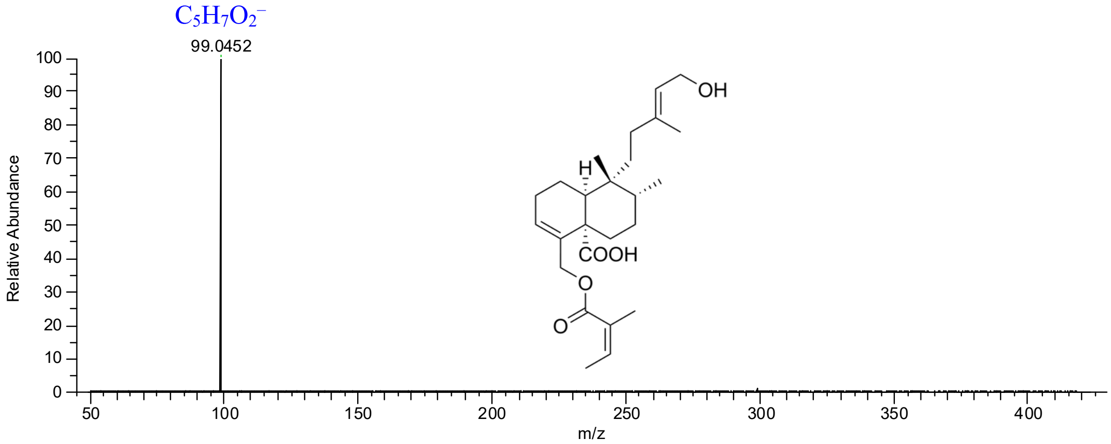

Figure S14. HR-ESI$^-$-MS/MS spectrum of solidagoic acid L (**1**) with a normalized HCD collision energy of 20%. Precursor ion: *m/z* 417.2644 [M−H]$^-$, $C_{25}H_{37}O_5^-$.

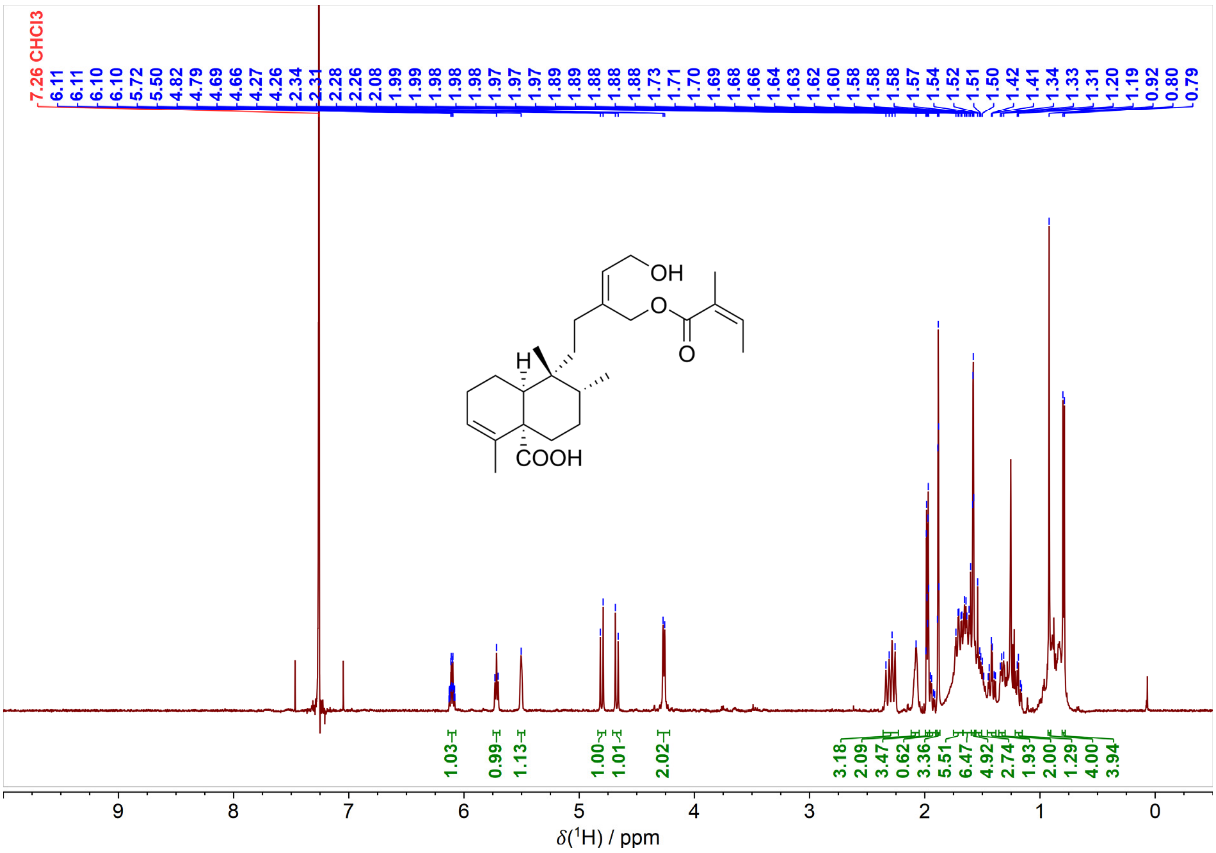

Figure S15. ^{1}H NMR spectrum of solidagoic acid M (**2**) (500 MHz, $CDCl_3$).

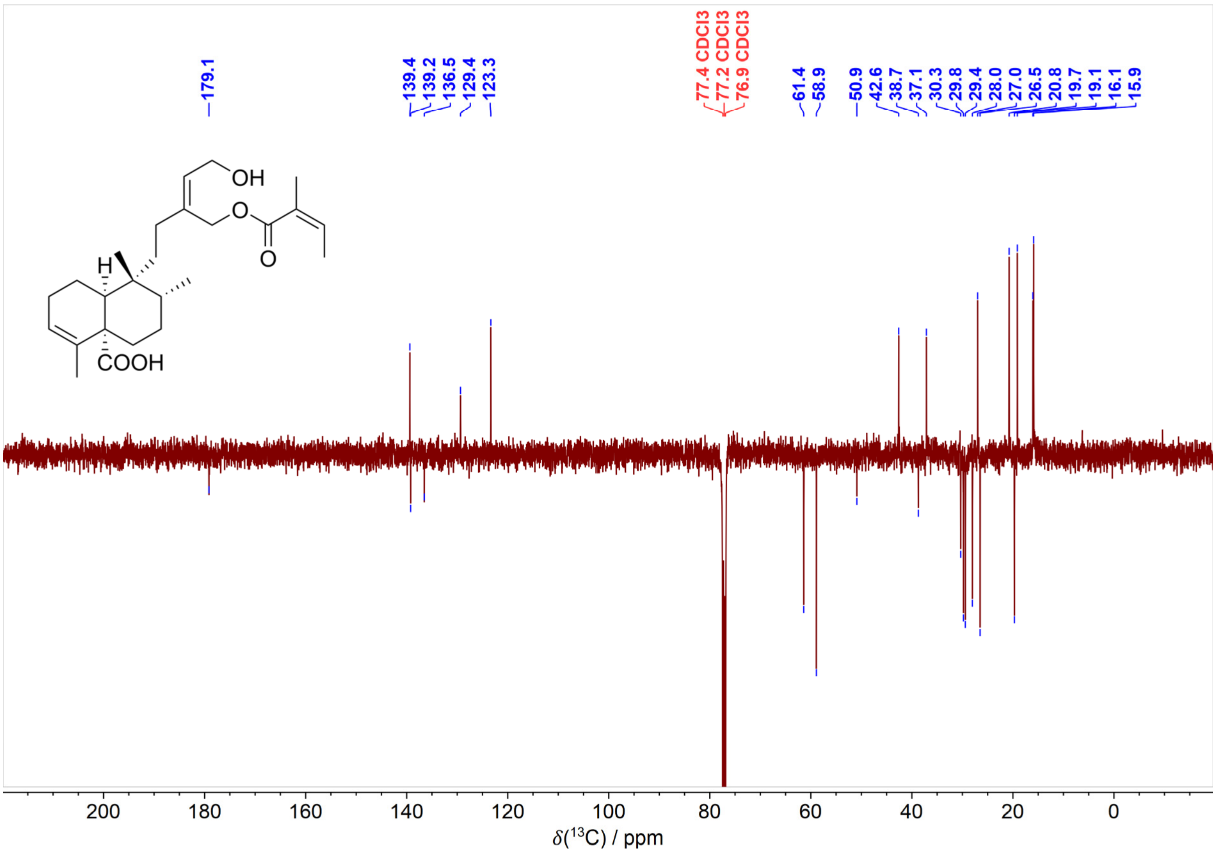

Figure S16. ^{13}C DEPTQ NMR spectrum of solidagoic acid M (**2**) (126 MHz, $CDCl_3$).

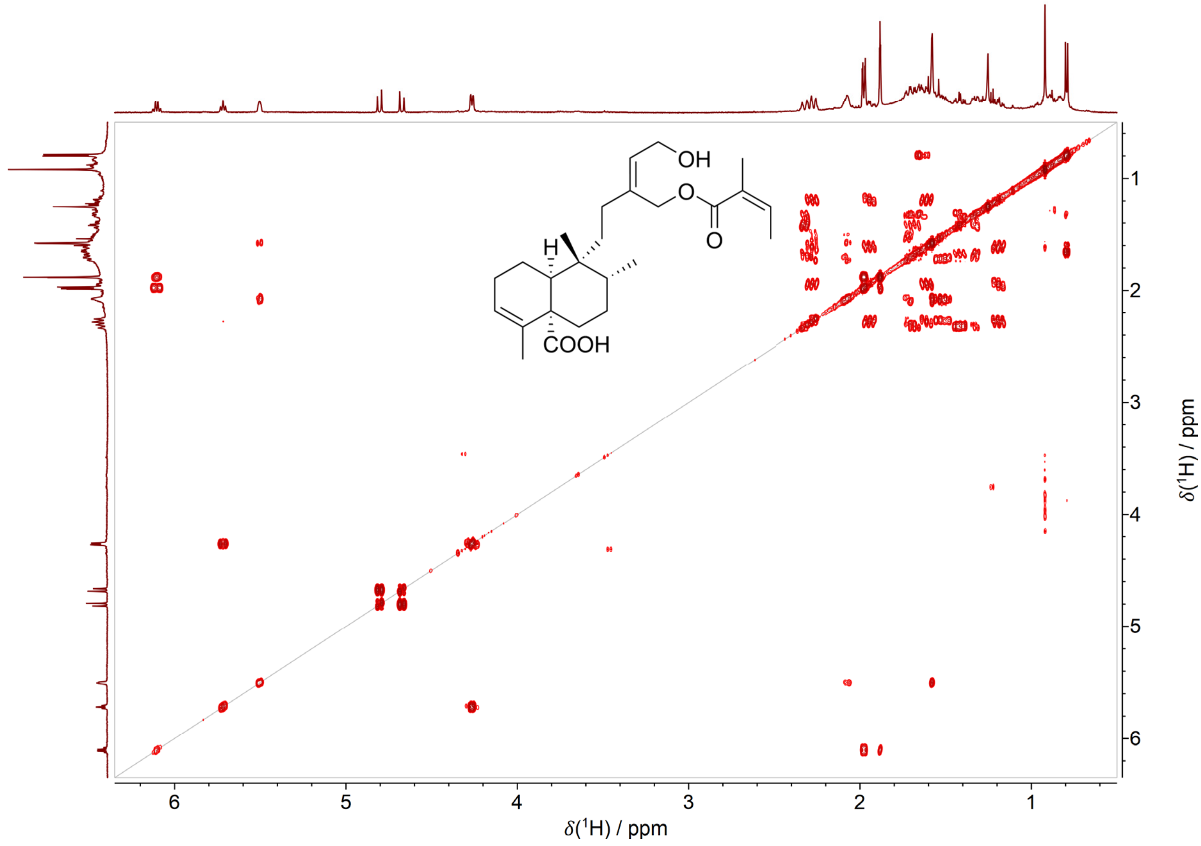

Figure S17. ^{1}H–^{1}H COSY NMR spectrum of solidagoic acid M (**2**) (500 MHz, $CDCl_3$).

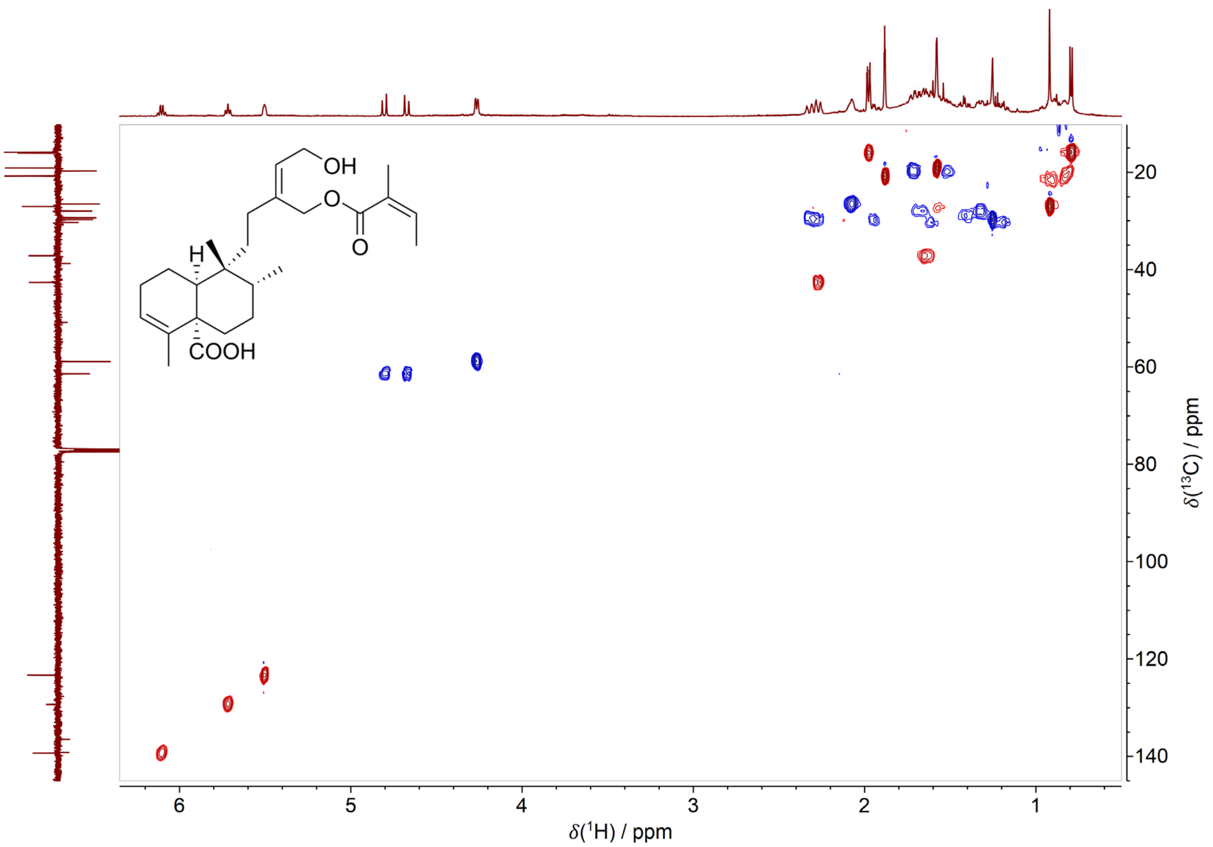

Figure S18. ^{1}H–^{13}C edHSQC NMR spectrum of solidagoic acid M (**2**) (500/126 MHz, $CDCl_3$).

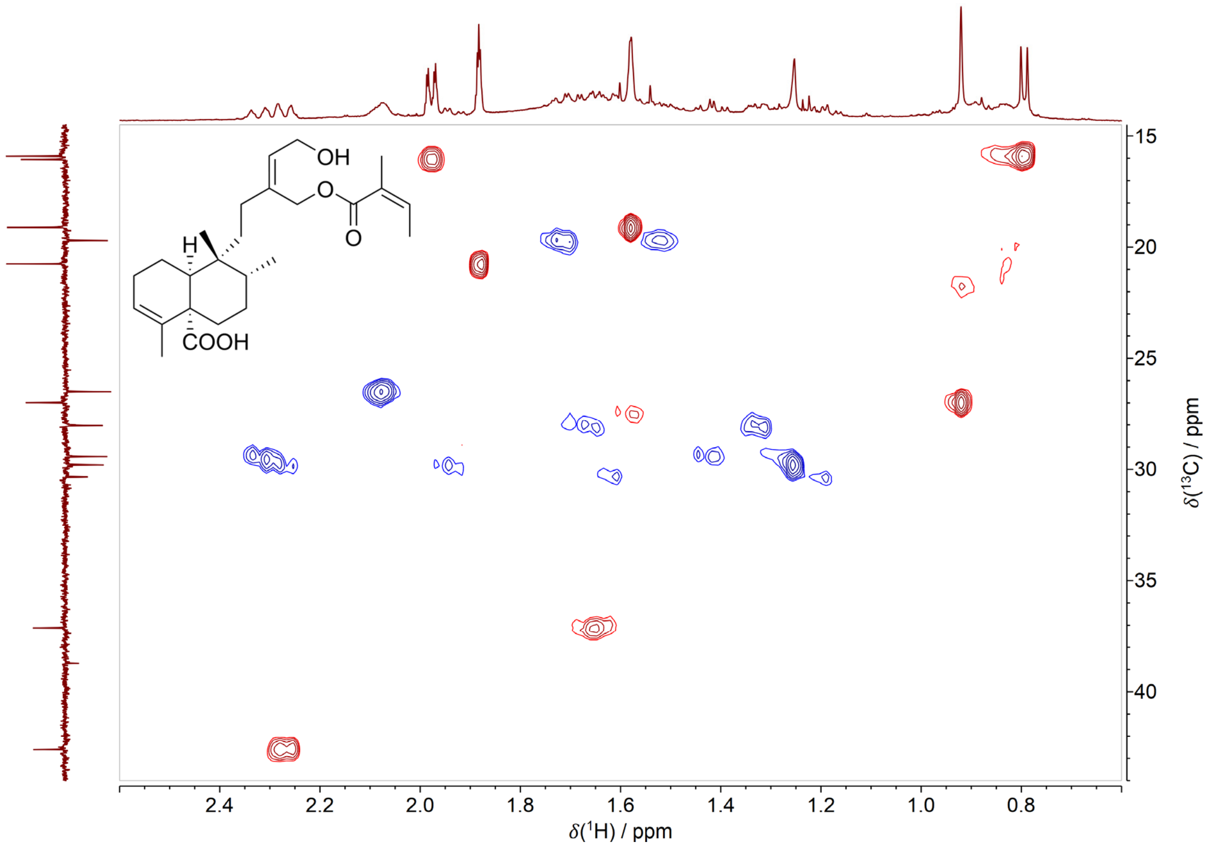

Figure S19. ^{1}H–^{13}C edHSQC NMR spectrum of solidagoic acid M (**2**) (500/126 MHz, $CDCl_3$) – aliphatic region (δ_C 44.0–14.5).

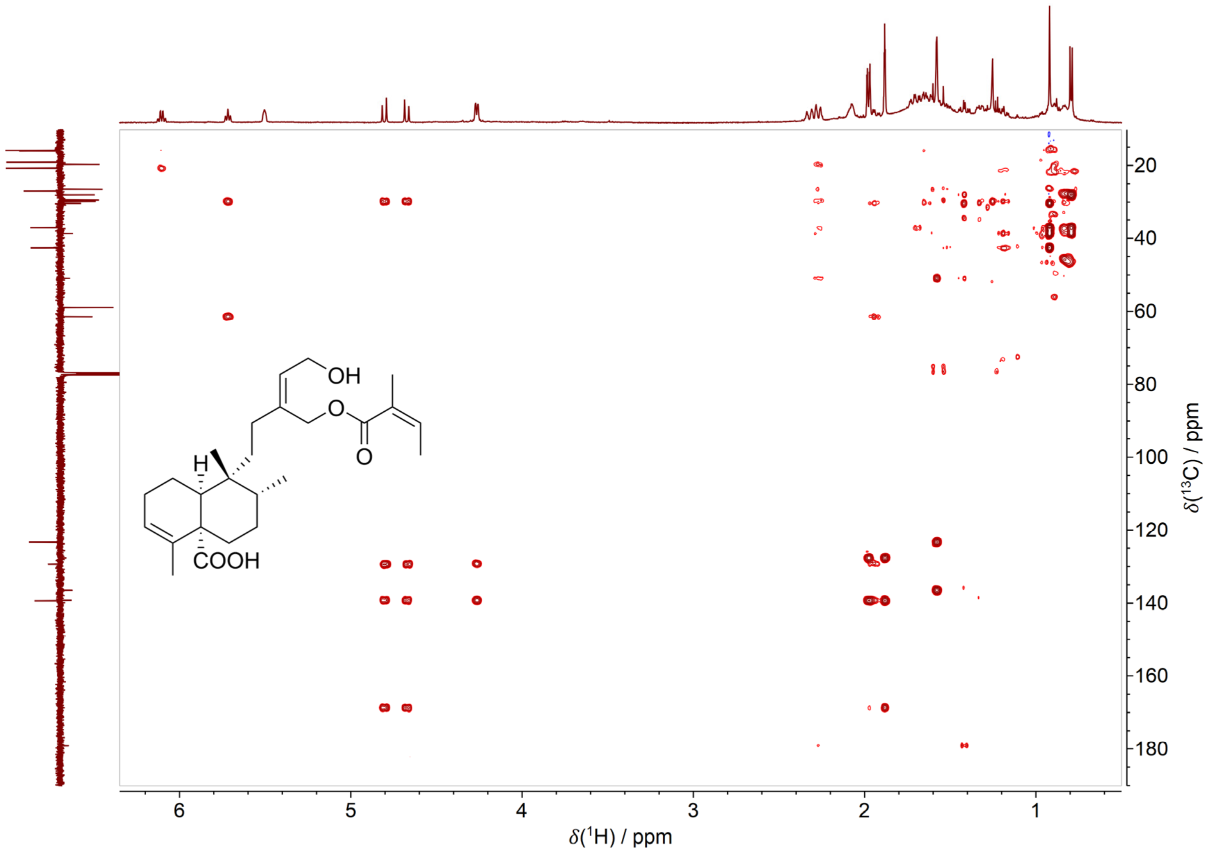

Figure S20. ^{1}H–^{13}C HMBC NMR spectrum of solidagoic acid M (**2**) (500/126 MHz, $CDCl_3$).

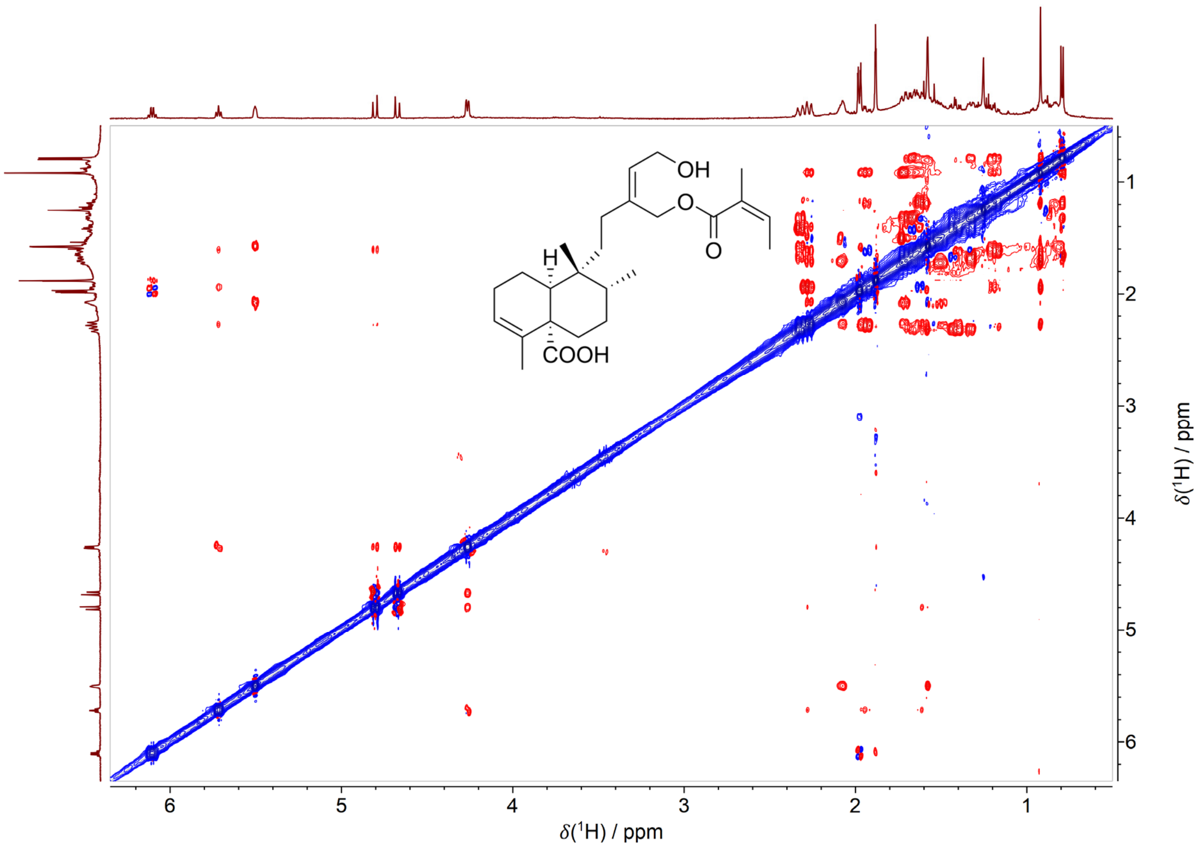

Figure S21. ^{1}H–^{1}H ROESY NMR spectrum of solidagoic acid M (**2**) (500 MHz, $CDCl_3$).

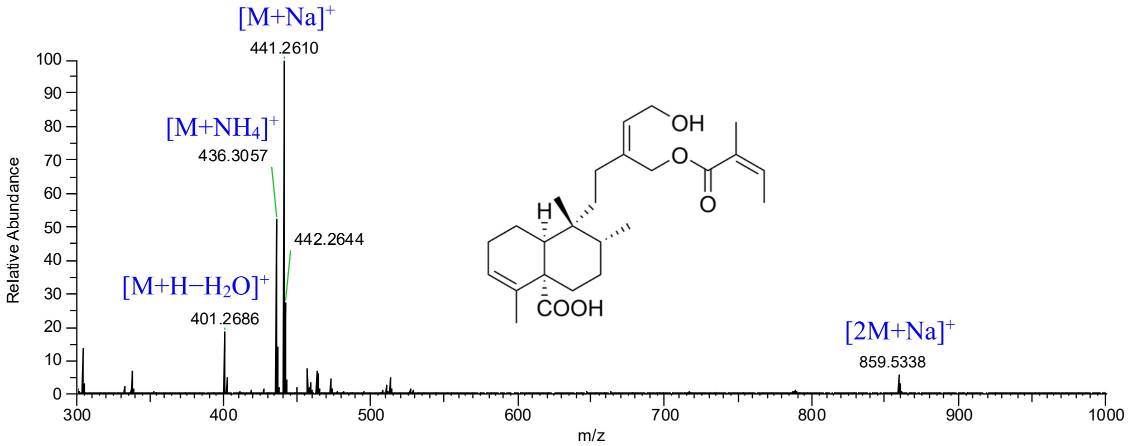

Figure S22. HR-ESI$^+$-MS spectrum of solidagoic acid M (**2**), *m/z* 441.2610 $[M+Na]^+$ (calculated for $C_{25}H_{38}O_5Na^+$, *m/z* 441.2612 $[M+Na]^+$, error: −0.3 ppm).

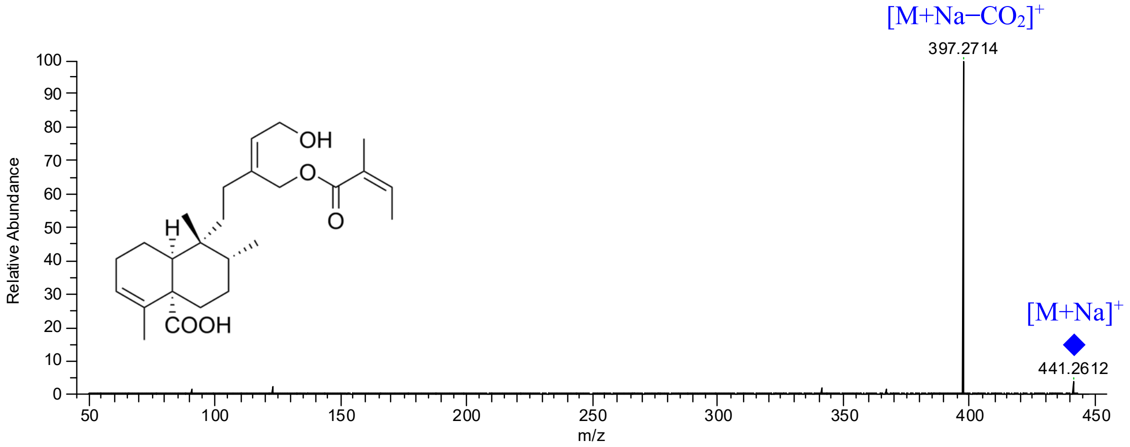

Figure S23. HR-ESI$^+$-MS/MS spectrum of solidagoic acid M (**2**) with a normalized HCD collision energy of 25%. Precursor ion: *m/z* 441.2612 $[M+Na]^+$, $C_{25}H_{38}O_5Na^+$.

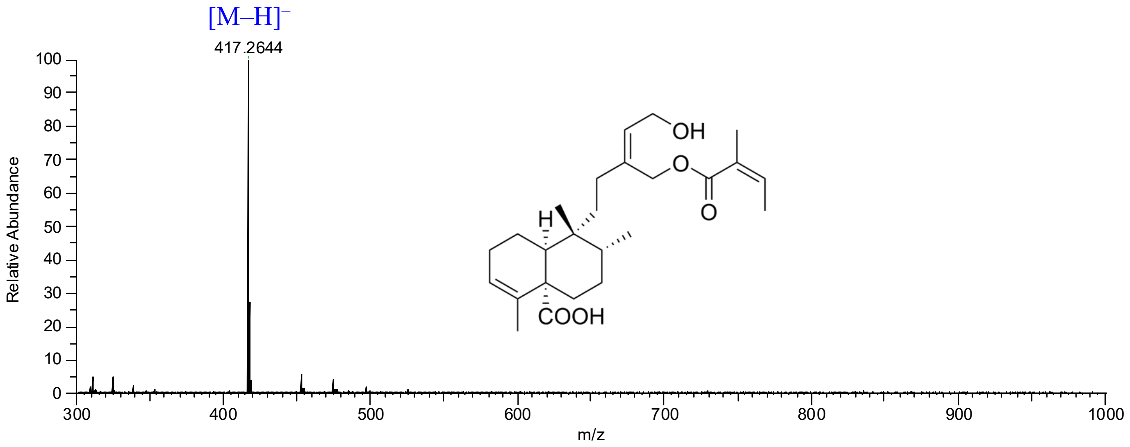

Figure S24. HR-ESI$^-$-MS spectrum of solidagoic acid M (**2**), *m/z* 417.2644 $[M-H]^-$ (calculated for $C_{25}H_{37}O_5^-$, *m/z* 417.2647 $[M-H]^-$, error: −0.6 ppm).

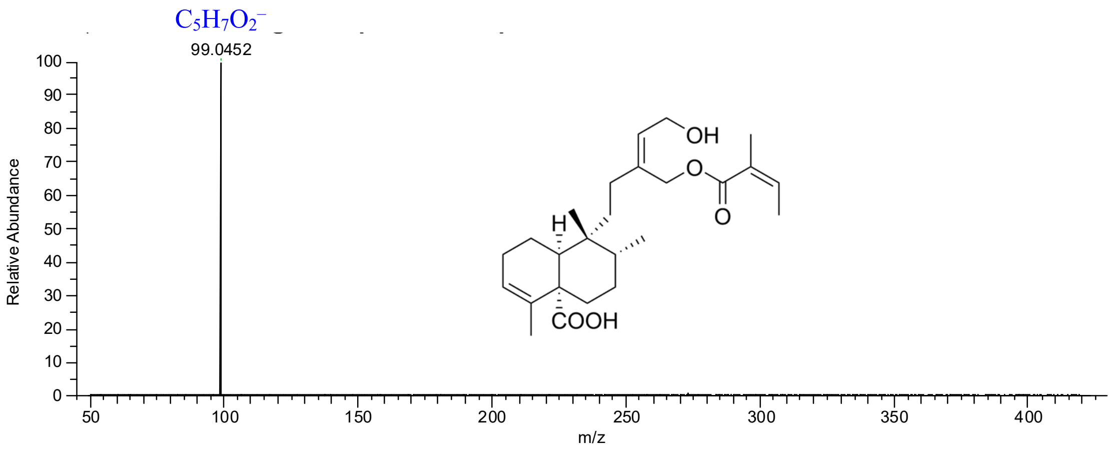

Figure S25. HR-ESI$^-$-MS/MS spectrum of solidagoic acid M (**2**) with a normalized HCD collision energy of 20%. Precursor ion: *m*/*z* 417.2644 [M–H]$^-$, $C_{25}H_{37}O_5^-$.
